# EphA2 contributes to disruption of the blood-brain barrier in cerebral malaria

**DOI:** 10.1371/journal.ppat.1008261

**Published:** 2020-01-30

**Authors:** Thayer K. Darling, Patrice N. Mimche, Christian Bray, Banlanjo Umaru, Lauren M. Brady, Colleen Stone, Carole Else Eboumbou Moukoko, Thomas E. Lane, Lawrence S. Ayong, Tracey J. Lamb

**Affiliations:** 1 Department of Pathology, University of Utah, Salt Lake City, UT, United States of America; 2 Department of Pediatric Infectious Diseases, Emory University School of Medicine, Atlanta, GA, United States of America; 3 Malaria Research Unit, Centre Pasteur du Cameroun, Yaoundé, Cameroon; 4 Department of Biological Sciences, University of Douala, Douala, Cameroon; Nanyang Technological University, SINGAPORE

## Abstract

Disruption of blood-brain barrier (BBB) function is a key feature of cerebral malaria. Increased barrier permeability occurs due to disassembly of tight and adherens junctions between endothelial cells, yet the mechanisms governing junction disassembly and vascular permeability during cerebral malaria remain poorly characterized. We found that EphA2 is a principal receptor tyrosine kinase mediating BBB breakdown during *Plasmodium* infection. Upregulated on brain microvascular endothelial cells in response to inflammatory cytokines, EphA2 is required for the loss of junction proteins on mouse and human brain microvascular endothelial cells. Furthermore, EphA2 is necessary for CD8+ T cell brain infiltration and subsequent BBB breakdown in a mouse model of cerebral malaria. Blocking EphA2 protects against BBB breakdown highlighting EphA2 as a potential therapeutic target for cerebral malaria.

## Author summary

Malaria is a disease caused by transmission of the mosquito-borne *Plasmodium* parasite that remains a severe global public health issue. Advancements in parasite control measures such as prevention, treatment, and surveillance have reduced the incidence of malaria worldwide. However, current reports indicate that progress towards reducing global malaria cases and deaths in recent years has stalled. Therefore, it is imperative that we continue to explore new therapeutic avenues that can synergize with existing treatment methods. In particular, there is currently no adjunctive treatment available for cerebral malaria which is a serious complication of *Plasmodium* infection characterized by blood-brain barrier breakdown. Here, we have identified that a receptor EphA2 is required for the breakdown of the blood-brain barrier during *Plasmodium* infection in mice. We found that expression of this receptor is critical for inducing brain inflammation, recruiting immune cells to the brain, and disruption brain endothelial cell junctions. Inhibiting activation of this receptor using two different treatment approaches also prevented blood-brain barrier breakdown in mice. Thus, along with identifying a new molecule critical for cerebral malaria in mice we also provide a basis for exploring this receptor as a novel therapeutic target in human cerebral malaria in the future.

## Introduction

Cerebral malaria (CM) is a severe manifestation of infection with the *Plasmodium falciparum* (*Pf*) parasite and has a 20% fatality rate[1]. Presenting as a plethora of neurological symptoms that lead to coma, pediatric CM is a complex disease that has been shown to involve alterations to, and breakdown of, the blood-brain barrier (BBB)[2–5]. This is thought to result from vascular activation in response to sequestration of *Plasmodium-*infected red blood cells (pRBCs) on the endothelium via adhesion molecules that include endothelial protein C receptor (EPCR)[6] and intercellular adhesion molecule 1 (ICAM-1)[7, 8]. Infection of mice with *Plasmodium berghei* ANKA (*Pb*A) has been used to demonstrate the importance of inflammatory cytokines such as interferon-γ (IFN-γ)[9] and tumor necrosis factor-β, also known as lymphotoxin-α (LT-α)[10], in the development of experimental cerebral malaria (ECM), a disease that shares several key features with human CM[11, 12]. Inflammation in ECM is T cell-mediated with CD8+ T cells playing a critical role in breakdown of the BBB[13–16]. However, apoptosis of brain endothelial cells does not appear to be sufficient to cause significant disruption of the barrier[15, 17]. The molecular mechanisms underlying BBB breakdown during *Plasmodium* infection are poorly understood, but the disruption of endothelial junctions is thought to be instrumental in this pathophysiological process.

Activation of receptor tyrosine kinases has been previously shown to play a role in endothelial junction disruption[18] and barrier integrity during ECM which can be maintained by global inhibition of the receptor tyrosine kinase family[17]. However, therapeutic potential of this observation is limited by the simultaneous inhibition of receptor tyrosine kinases that are also involved in mounting an effective immune response[19] which could detrimentally affect control of *Plasmodium* infection. Identification of the major receptor tyrosine kinases necessary for junction disruption during CM is required to capitalize on strategies to specifically target receptor tyrosine kinases for therapeutic benefit.

Erythropoietin-producing hepatocellular (Eph) receptors constitute the largest family of receptor tyrosine kinases in humans and are ubiquitously expressed in nearly all tissues, including the brain[20] in both mice and humans. There are nine different functional EphA receptors in the mouse and human genome (EphA1-EphA9) that have the ability to interact with five membrane-bound Eph receptor interacting (ephrin) ligands (ephrin-A1-ephrin-A5) with varying affinities[21]. The unique expression patterns of EphA receptors and ephrin-A ligands in different tissues and cell types allows for functional specificity, and EphA-ephrin-A binding between cells canonically leads to events such as cellular migration, adhesion, and changes in cellular morphology[22]. As the interaction between EphA receptors and membrane-bound ephrin-A ligands is of high-affinity, this initial binding event will often lead to strong adhesion between the two cells involved. This can progress to either extended adhesion or repulsion and separation of the two cell surfaces once signaling pathways are propagated depending on the context[21]. As a prime example of these multifunctional receptors, one particular EphA family member, EphA2, can be utilized by CD8+ T cells for chemotaxis[23] and adhesion[24]. Additionally, EphA2 has also been previously shown to be instrumental in the disassembly of both tight and adherens junction protein complexes on endothelial cells reducing cell-cell contact[25, 26]. Given that EphA receptors play a role in regulating both brain endothelial junction formation and immune cell migration and adhesion, processes highly relevant to the development of CM, here we have investigated the role of EphA receptors in malaria-associated BBB breakdown. We found that EphA2 is upregulated on both human and mouse primary brain microvascular endothelial cells in response to tumor necrosis factor family cytokines. In mice, EphA2 is upregulated by LT-α, a cytokine required for BBB breakdown in *Pb*A infection[10, 27]. EphA2 deficient mice exhibit significantly improved survival in comparison to EphA2 sufficient mice, likely as a result of reduced CD8+ T cell brain infiltration and inflammation along with maintenance of brain microvascular endothelial cell junctions. Collectively, this results in enhanced BBB integrity. Interestingly, brain EphA2 upregulation is a unique feature of infection with the ECM-causing *Pb*A strain and does not occur upon infection with strains that do not cause ECM. This suggests EphA2 upregulation on brain microvascular endothelial cells is critical for *Plasmodium*-associated cerebral pathology. Blocking the interaction between EphA2 and its cognate ephrin-A ligands increases the integrity of the BBB during ECM which demonstrates a rationale for exploring EphA2 antagonism as a novel therapeutic strategy for maintaining BBB integrity during CM.

## Results

### EphA2 is required for blood-brain barrier breakdown and the development of ECM

**Fig 1:**
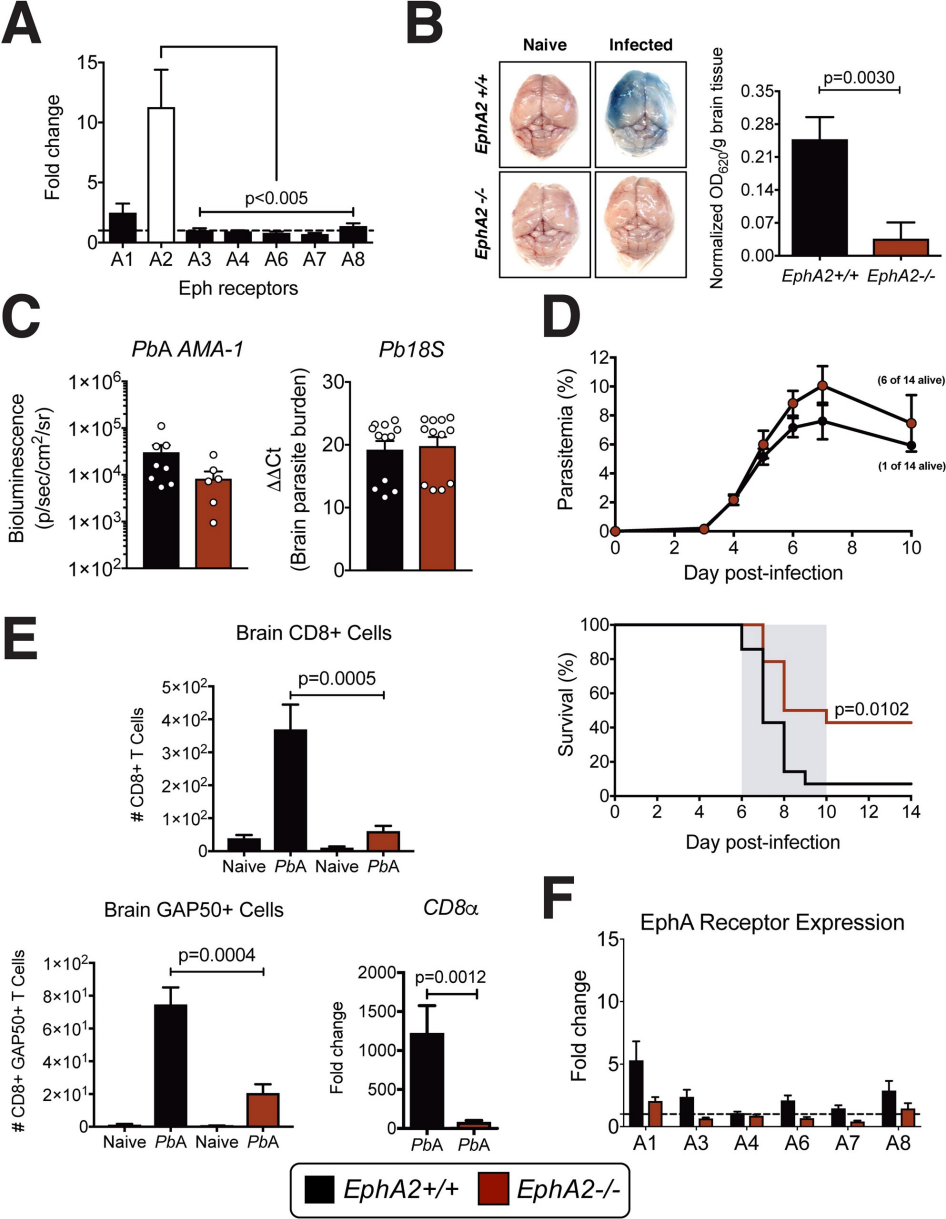
EphA2 is required for blood-brain barrier breakdown and the development of ECM. **(A)** Transcription of EphA receptors relative to naïve mice (dashed line) in whole brains of C57BL/6J mice (n = 16/group) at day 6 post-infection with *Pb*A. **(B)** Brain permeability in *EphA2-/-* and littermate control mice injected intravenously with 1% Evan’s Blue at day 6 post-infection with *Pb*A. Representative images and quantification of dye extracted from whole brains is shown (n = 9-10/group). OD values are normalized to naïve mice from each respective group. **(C)** Quantification of sequestered *Pb*A schizonts expressing luciferase under the *AMA-1* promoter (n = 7-8/group) and 18S parasite DNA transcript (n = 12/group) in whole brains of *EphA2-/-* and littermate control mice at day 6 post-infection. **(D)** Peripheral parasitemia and survival of *EphA2-/-* and littermate control mice infected with *Pb*A (n = 14/group). **(E)** Total CD8+ T cells (top left; n = 12-14/group), GAP50+CD8+ T cells (bottom left; n = 7-10/group), and transcription of CD8α relative to naïve mice (dashed line) (bottom right; n = 9/group) in brains of *EphA2-/-* and littermate control mice at day 6 post-infection with *Pb*A. Naïve and *Pb*A-infected groups are significantly different within each genotype. **(F)** Transcription of EphA receptors relative to naïve mice (dashed line) in whole brains of *EphA2-/-* and littermate control mice (n = 12-13/group) at day 6 post-infection with *Pb*A. Bars in all graphs represent the mean ± SEM. Statistical analyses: Kruskal-Wallis and Dunn’s multiple comparisons tests (A), Mann-Whitney test (B, C, E) and Log-rank Mantel-Cox test (D). Only statistically significant (p<0.05) values are shown unless otherwise noted in the legend. Figures represent combined data from 2 (B, C-left panel, D, E-bottom left panel), 3 (C-right panel, E-top and bottom right panels, F), or 4 (A) independent experiments.

**Fig 2:**
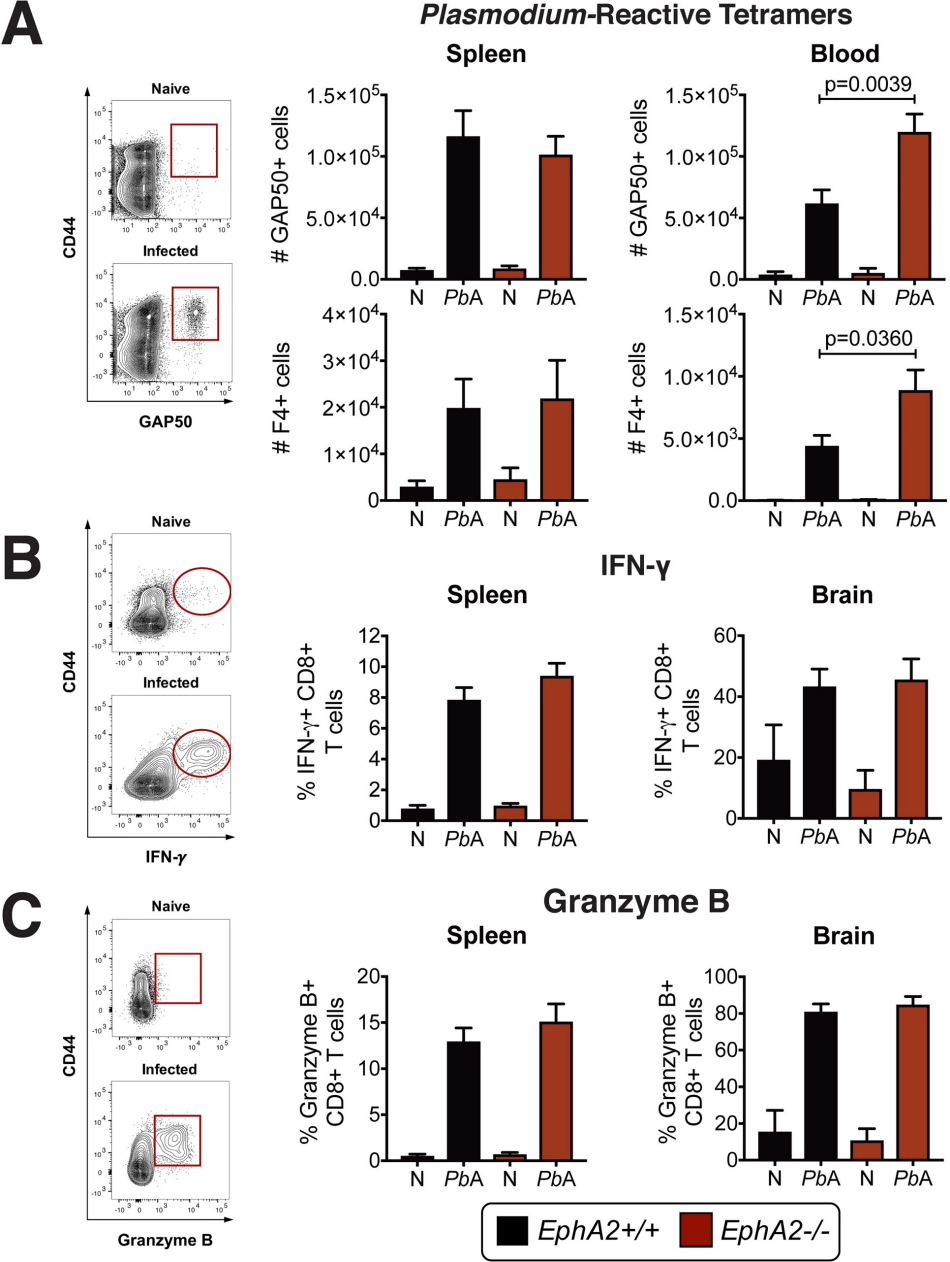
CD8+ T cell expansion and functionality is not affected by EphA2 deficiency. **(A)** Representative staining and total numbers of *Plasmodium* GAP50-reactive (n = 11-18/group) and F4-reactive (n = 8-9/group) CD8+T cells present in the spleen (left) and bloodstream (right) of *EphA2-/-* and littermate control mice at day 6 post-infection with *Pb*A compared to naïve (N) mice (n = 4-8/group). Naïve and *Pb*A-infected groups are significantly different within each genotype for all graphs except bottom left. **(B-C)** Frequency of IFN-γ+ **(B)** or Granzyme B+ **(C)** CD8+ T cells in the spleen (n = 10-15/group) and brain (n = 7-10/group) of *EphA2-/-* and littermate control mice at day 6 post-infection with *Pb*A compared to naïve (N) mice (n = 4/group). Naïve and *Pb*A-infected groups are significantly different within each genotype for all graphs except *EphA2+/+* in B-right panel. Bars in all graphs represent the mean ± SEM. Statistical analysis: Mann-Whitney test (A-C). Only statistically significant (p<0.05) values are shown unless otherwise noted in the legend. Figures represent combined data from 2 (A-bottom left and right panels, B-right panel, C-right panel), 3 (A-top right panel, B-left panel, C-left panel), or 4 (A-top left panel) independent experiments.

Given the known roles of EphA receptors in mediating cellular interactions in the brain, we first determined if members of the EphA family of receptors were modulated at the onset of ECM. A comparison of the transcriptional profile of the EphA receptor subfamily in whole brain tissue isolated from mice infected with *Pb*A revealed a significant upregulation of *EphA2* transcript at day 6 post-infection (**Fig 1A**) at the onset of ECM symptoms along with a slight upregulation of *EphA1* transcript. In the absence of EphA2, *Pb*A-infected mice maintained an intact BBB (**Fig 1B**) at the onset of ECM in comparison to EphA2 sufficient mice. However, there was no significant difference in the parasite burden in *EphA2-/-* and *EphA2+/+* mice when assessing the levels of parasites by *Pb*A luciferase expression in the brain (**Fig 1C, left, and S1A Fig**), parasite mRNA in the brain (**Fig 1C, right**), or peripheral parasitemia (**Fig 1D, top**). However, *EphA2-/-* mice did have significantly less *Pb*A accumulation in the spleen and liver compared to *EphA2+/+* control mice (**S1B Fig**). The maintained BBB integrity observed in *Pb*A-infected *EphA2-/-* mice translated into significantly improved survival compared to *EphA2+/+* control mice (**Fig 1D, bottom**). Maintenance of BBB integrity and improved survival were also associated with a failure of CD8+ T cells, including *Plasmodium*-reactive GAP50+CD8+ T cells[28], to accumulate in the cerebral microvasculature (**Fig 1E**). No compensatory transcriptional upregulation of other EphA receptors occurred in the brains of *EphA2-/-* mice infected with *Pb*A **(Fig 1F)** indicating that the upregulation of EphA2 in *Plasmodium* infection is independently regulated from other EphA receptors. Using the previously identified CD8+ T cell *Plasmodium*-reactive tetramers GAP50 and F4[28, 29] and analyzing IFN-γ and Granzyme B which are essential molecules for ECM development[13, 16], we found that the absence of CD8+ T cells observed in the brains of *EphA2-/-* mice was not due to a defect in the splenic expansion or egress of highly functional *Plasmodium*-reactive CD8+ T cells (**Fig 2A–2C**) but rather on a failure of CD8+ T cells to accumulate in the brain microvasculature in the absence of EphA2. *EphA2-/-* mice had significantly higher numbers of circulating *Plasmodium*-reactive CD8+ T cells in their bloodstream (**Fig 2A**) demonstrating that mice are able to mount a robust CD8+ T cell response against the parasite in the absence of EphA2.

### *EphA2* is upregulated in human and mouse primary brain endothelial cells in response to inflammatory cytokines

**Fig 3:**
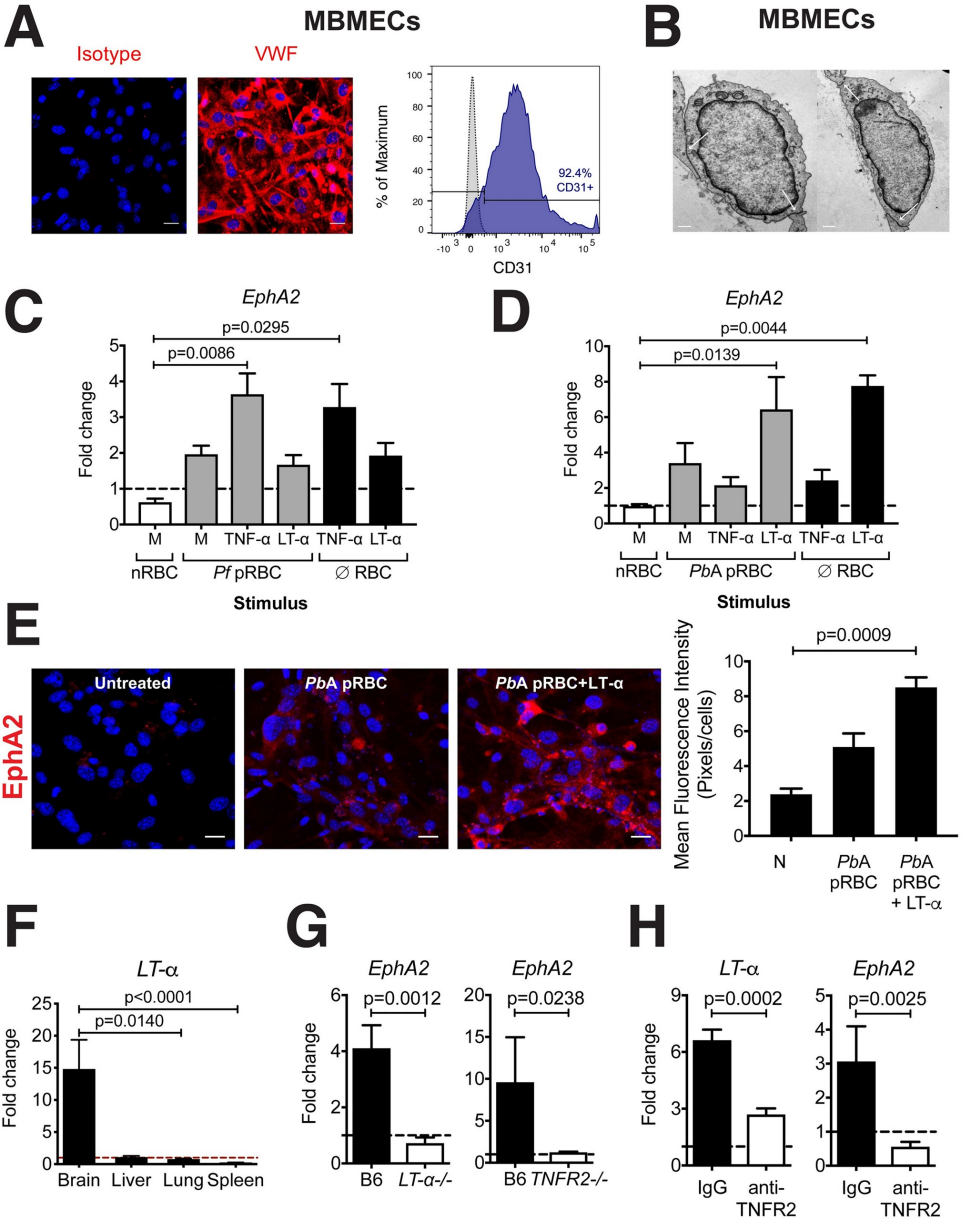
*EphA2* is upregulated in human and mouse primary brain endothelial cells in response to inflammatory cytokines. Representative data showing von Willebrand factor (VWF) immunofluorescence staining (cell nuclei stained with DAPI-blue) **(A, left)**, CD31 flow cytometry staining (gray histogram: isotype control, blue histogram: anti-CD31) **(A, right)**, and transmission electron microscopy **(B)** of cultured MBMECs isolated from C57BL/6J mice. White arrows in **(B)** indicate endothelial cell contact points. Scale bars represent 25μm **(A)** and 0.5μm **(B)**. **(C-D)** *EphA2* transcription in human **(C)** and mouse **(D)** BMECs incubated for 24 hours with naïve RBC lysates (nRBC), *P*. *falciparum* 3D7-infected RBC lysates (*Pf* pRBC), *Pb*A-infected RBC lysates (*Pb*A pRBC), or no RBC lysate (∅ RBC) plus human **(C)** or mouse **(D)** LT-α, TNF-α, or media (M) (n = 3–6 endothelial preparations/group). Values are normalized to untreated cells. **(E)** Immunofluorescence images and fluorescence quantification of EphA2 (red) on MBMECs unstimulated or stimulated with *Pb*A-infected RBC lysates (*Pb*A pRBC) in the presence of absence of LT-α for 24 hours. Cell nuclei stained with DAPI (blue). Scale bars represent 25μm. Images representative of two endothelial preparations. **(F)** *LT-α* transcription relative to naïve mice (dashed line) in whole brains, livers, lungs, and spleens of C57BL/6J mice (n = 8-9/group) at day 6 post-infection with *Pb*A. **(G)** *EphA2* transcription relative to naïve mice (dashed line) in brains of *LT-α-/-* (n = 7) and *TNFR2-/-* (n = 3) mice at day 6 post-infection with *Pb*A compared to wild-type C57BL/6J mice (n = 6–11). **(H)** *LT-α* and *EphA2* transcription relative to naïve mice (dashed line) in brains of isotype control (n = 9) and anti-TNFR2 (n = 8) treated mice at day 6 post-infection with *Pb*A. Bars in all graphs represent the mean ± SEM. Statistical analyses: Kruskal-Wallis and Dunn’s multiple comparisons tests (C, D, E, F) and Mann-Whitney test (G, H). Only statistically significant (p<0.05) values are shown. Figures represent combined data from 1 (G-right panel), 2 (E, F, G-left panel, H), or 3 (C, D) independent experiments.

EphA2 expression on endothelial cells has previously been associated with impairment of junction formation[26]. Since the majority of pRBCs[15] and CD8+ T cells[30] are known to adhere to the brain microvascular endothelium on the luminal surface of blood vessels during ECM, we sought to determine the impact of *Pb*A infection specifically on endothelial-expressed EphA2. We confirmed that EphA2 is upregulated at the protein level in brains of *Pb*A-infected wild-type mice and colocalizes primarily with the brain vasculature (**S2 Fig**). To determine if the pRBCs or inflammatory cytokines were responsible for this observed upregulation of EphA2 on brain endothelial cells, we isolated both human primary brain microvascular endothelial cells (HBMECs) and mouse primary brain microvascular endothelial cells (MBMECs) as confirmed by staining for the endothelial-specific markers von Willebrand factor (VWF) **(Fig 3A, left)** and CD31 **(Fig 3A, right)** and the formation of cell-cell contacts by transmission electron microscopy **(Fig 3B)**. We cultured the cell monolayers with their respective parasites (*Pf* pRBCs with HBMECs and *Pb*A pRBCs with MBMECs) along with the inflammatory cytokines tumor necrosis factor-alpha (TNF-α) and LT-α which are known to be produced in the brain during *Plasmodium* infections. *EphA2* expression was significantly increased in HBMECs pulsed with TNF-α (**Fig 3C**). The addition of *Plasmodium falciparum*-infected red blood cells (*Pf* pRBC) had no synergistic effect which supports previous studies showing upregulation of *EphA2* in human microvascular endothelial cells and monocytes by TNF-α[31, 32]. On the other hand, MBMECs upregulated *EphA2* primarily in response to LT-α (**Fig 3D and 3E**). This result is biologically significant because production of TNF-β/LT-α by non-hematopoietic cells in *Pb*A infection is required for ECM development[10]. The *Pb*A-associated upregulation of *EphA2* mRNA appeared specific to the brain tissue as transcript levels remained essentially unchanged in liver and lung tissues (**S3A and S3B Fig**). Furthermore, *Plasmodium*-reactive CD8+ T cell accumulation in pulmonary tissue was identical in the presence or absence of EphA2 (**S3C and S3D Fig**). This brain-specific *EphA2* upregulation likely results from the fact that *LT-*α is only upregulated in the brain during ECM with little to no increase in *LT-*α mRNA levels in the liver, lung, or spleen at day 6 post-infection with *Pb*A **(Fig 3F)**. In agreement with these observations, *EphA2* transcript was not upregulated in the brains of LT-α deficient and tumor necrosis factor receptor 2 (TNFR2) deficient mice infected with *Pb*A in contrast with wild-type mice (**Fig 3G**). Additionally, treatment of *Pb*A-infected C57BL/6J mice with an anti-TNFR2 blocking antibody resulted in significantly reduced mRNA levels of both *LT-*α and *EphA2* in the brain in comparison to isotype control treated mice **(Fig 3H)**. These data provide further support for the role of LT-α in inducing *EphA2* upregulation in the brains of mice at the onset of ECM.

### Ephrin-A ligands are upregulated during *Plasmodium* infection

**Fig 4:**
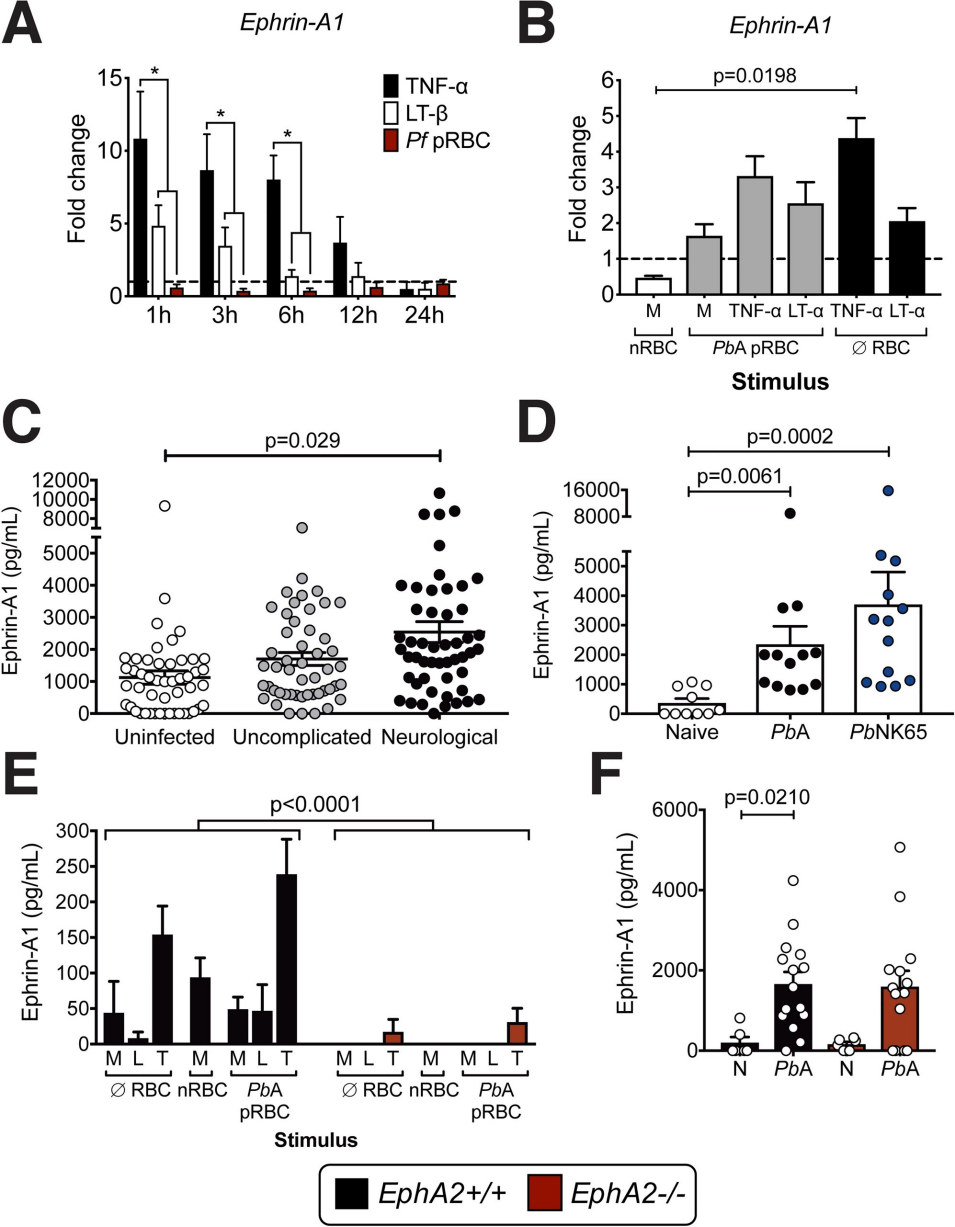
Ephrin-A ligands are upregulated and cleaved as a result of *Plasmodium* infection. **(A)** *Ephrin-A1* ligand transcription in HBMECs incubated for 1–24 hours with human TNF-α, LT-β, or *P*. *falciparum* 3D7-infected RBC lysates (*Pf* pRBC) (n = 3 endothelial preparations/group/time point). Values are normalized to untreated cells. Asterisk indicates p<0.05. **(B)** *Ephrin-A1* ligand transcription in MBMECs incubated for 24 hours with naïve RBC lysates (nRBC), *Pb*A-infected RBC lysates (*Pb*A pRBC), or no RBC lysate (∅ RBC) plus mouse LT-α, TNF-α, or media (M)) (n = 3 endothelial preparations/group). **(C)** Levels of soluble ephrin-A1 ligand in the plasma of children living in an area in Cameroon endemic for *P*. *falciparum* malaria. Patients were categorized by admission to the hospital for neurological complications (n = 51), uncomplicated malaria (n = 50), or uninfected and presenting for routine pediatric tests (n = 49). Each dot represents an individual patient. **(D)** Levels of soluble ephrin-A1 ligand in the plasma of C57BL/6J mice infected with with *Pb*A (n = 13) or *Pb*NK65 (n = 13) at day 6 post-infection compared with naïve mice (n = 10). **(E)** Ephrin-A1 ligands released from MBMECs derived from *EphA2-/-* or littermate control mice and incubated for 24 hours with no RBC lysate (∅ RBC), naïve RBC lysate (nRBC), and *Pb*A-infected RBC lysate (*Pb*A pRBC) with the addition of media (M), recombinant mouse LT-α (L), or TNF-α (T) (n = 4 endothelial preparations). **(F)** Ephrin-A1 ligands in the plasma of *EphA2-/-* or littermate control mice at day 6 post-infection with *Pb*A (n = 14-15/group) compared to naïve mice (N) (n = 6/group). Bars in all graphs represent the mean ± SEM. Statistical analyses: Two-way ANOVA (A, E), Kruskal-Wallis and Dunn’s multiple comparisons tests (B, D, F), and General linear modeling and Tukey’s pairwise comparison post-ANOVA (C). Only statistically significant (p<0.05) values are shown. Figures represent combined data from 3 (A, B, D, F), or 4 (E) independent experiments.

Ephrin-A ligands are the cognate ligands for Eph receptors and are expressed on a variety of tissues and cell types. Upon binding to Eph receptors, they can be cleaved by matrix metalloproteinases (MMPs) and a disintegrin and metalloproteinase domain-containing (ADAMs) proteins, a family of zinc proteases[33–36]. This occurs particularly in inflammatory environments[37, 38] resulting in the release of soluble ligand fragments into the bloodstream. As cell membrane-bound ephrin dimer complexes are thought to be a requirement to induce Eph receptor downstream signaling[39, 40], soluble ephrin ligands are not generally believed to activate Eph receptors although there is some evidence that this can occur[41]. As each Eph receptor, including EphA2, can promiscuously bind to several ephrin ligands (ephrin-A1-5) with varying affinities, we focused on the main high affinity ligand for EphA2, ephrin-A1, along with a secondary ligand for EphA2, ephrin-A5[42–44]. Like EphA2, ephrin-A1 ligand expression is known to be induced by TNF family members[45–47]. Therefore, we analyzed expression of *ephrin-A1* in HBMECs and MBMECs upon incubation with pRBCs, TNF-α, or LT-α alone or in varying combinations. We found that TNF-α was the primary driver of *ephrin-A1* transcription in both HBMECs and MBMECs with pRBCs having no apparent effect (**Fig 4A and 4B**). However, *P*. *falciparum* did significantly upregulate *ephrin-A1* ligand and *ephrin-A5* ligand expression on peripheral blood mononuclear cells (PBMCs) isolated from healthy individuals not previously exposed to malaria in a dose-dependent manner *in vitro* (**S4A and S4B Fig**). Cells upregulating these ephrin ligands included, but were not limited to, CD3+ T cells (**S4C and S4D Fig**). Transcription of *ephrin-A1* and *ephrin-A5* ligands was also modulated in splenic T cells isolated from *Pb*A-infected mice at day 5 post-infection prior to their egress from the spleen (**S4E Fig**).

Given this significant induction of *ephrin-A1* transcript in both mouse and human brain endothelial cells and leukocytes, we next tested whether soluble ephrin-A ligands are released into the bloodstream during *Plasmodium* infection. We detected soluble ephrin-A1 ligand in the plasma of *P*. *falciparum*-infected pediatric patients, where those patients experiencing neurological manifestations had a significantly higher concentration of soluble ephrin-A1 ligand compared to uninfected patients (F_2,149_ = 3.61; P = 0.029) when patient age and parasite burden were taken into consideration (Uninfected = 54.1±6.6 months with 0±0 parasites/μL blood; Uncomplicated = 72.3±6.5 months with 83,034±17,019 parasites/μL blood; Neurological = 46.1±6.0 months with 142,598±23,138 parasites/μL blood) (**Fig 4C**). Unlike ephrin-A1 ligand, there was no correlation between the presence of soluble EphA2 receptor in the plasma and neurological symptoms in the same cohort of pediatric patients (F_2,149_ = 1.6; P = 0.206) (**S4F Fig**). Soluble ephrin-A1 ligands were also detected in the plasma of C57BL/6J mice infected with *Plasmodium* (**Fig 4D**) at significantly higher levels than naïve mice. However, ephrin-A1 ligand was also highly prevalent in plasma of mice infected with a parasite strain related to *Pb*A, *Plasmodium berghei* NK65 (*Pb*NK65), which does not cause ECM but causes inflammation and pathology in other organs, particularly the lungs[48]. This data indicates that general inflammation is likely leading to increased levels of soluble ephrin-A1 ligand and that this phenomenon is not limited to neurological disease.

As the shedding of ephrin-A ligands is believed to occur subsequent to binding EphA receptors, we investigated if *EphA2-/-* MBMECs had a defect in the release of soluble ephrin-A1 ligand. We found that ephrin-A1 ligand was shed by *EphA2+/+* MBMECs primarily in response to TNF-α (**Fig 4E**). On the contrary, *EphA2-/-* MBMECs released little to no ephrin-A1 ligand suggesting that the presence of EphA2 on brain endothelial cells can mediate ephrin-A1 cleavage and release. This could potentially occur through interactions with ADAMs and MMPs which are modulated during *Plasmodium* infection **(S5A and S5B Fig)** and can cleave ephrin-A1 ligands[33–36, 49, 50]. However, there was no significant difference in levels of plasma ephrin-A1 ligand between *Pb*A-infected *EphA2-/-* and *EphA2+/+* mice (**Fig 4F**). This indicates that ephrin-A1 ligands can be shed in an EphA2-independent manner from other organ sites and cell types during infection, likely through interactions with other EphA receptors that ephrin-A1 is able to bind. Collectively, this data suggests that although ephrin-A ligand upregulation and shedding are induced during the inflammatory immune response to malaria, the modulation and expression of the membrane-bound receptor, EphA2, is likely more of a critical factor influencing susceptibility to ECM.

### EphA2 expression in the brain is a hallmark of pathogenesis in ECM

CD8+ T cell accumulation in the brain is necessary[51] but not sufficient[28] for the development of ECM. Previous studies have attributed CD8+ T cell participation in ECM in the brain microvasculature as dependent on MHC-I cross-presentation of malaria peptides, a phenomenon driven by IFN-γ. In addition to pMHC-I/TCR interactions, we hypothesized that a signal provided by EphA2 ligation is required to mediate BBB disruption.

**Fig 5:**
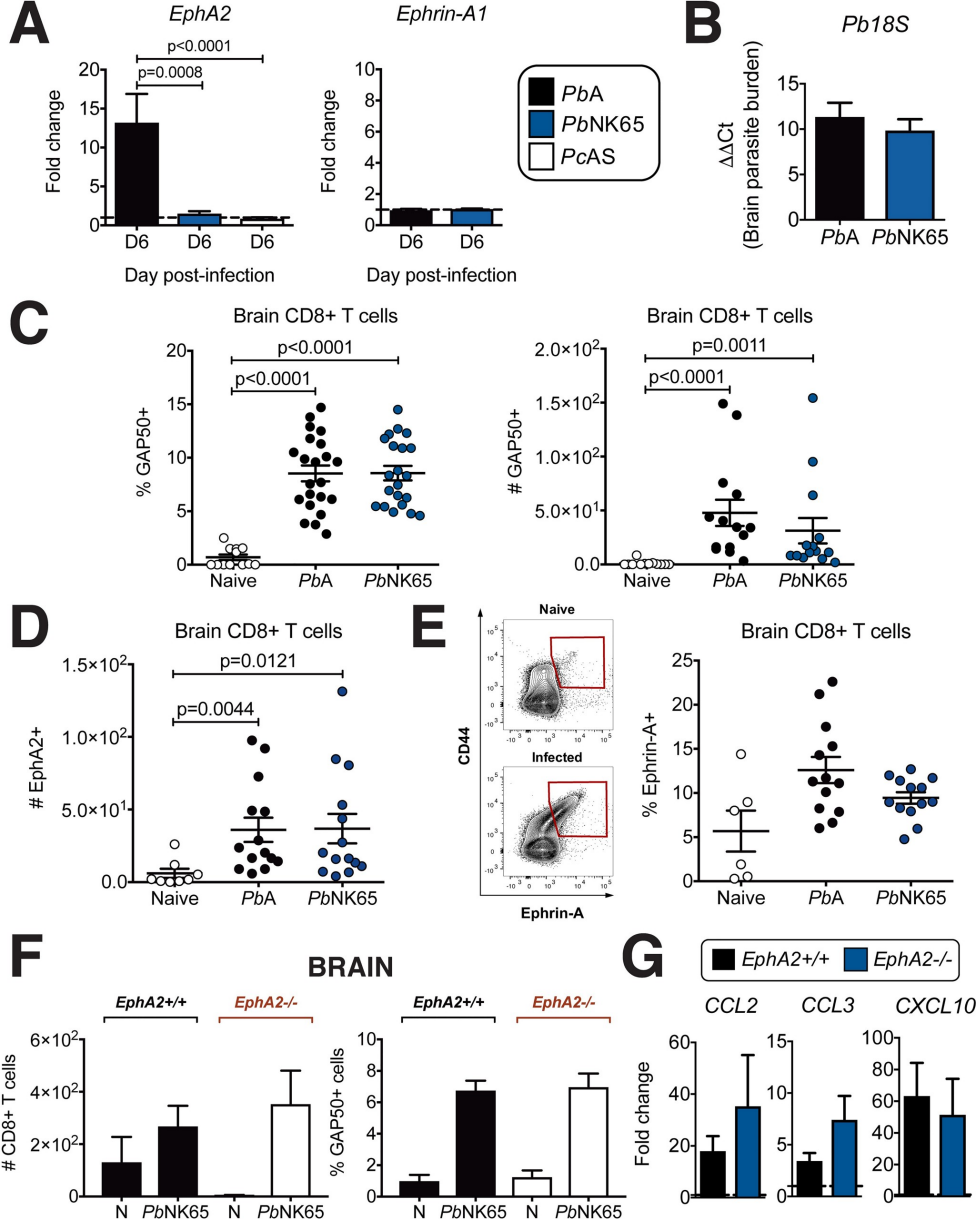
EphA2 is not required for trafficking of CD8+ T cells in the brain during *Pb*NK65 infection. **(A)** Transcription of *EphA2* (left) and *ephrin-A1* (right) in whole brain lysates of C57BL/6J mice infected with *Pb*A (n = 12), *Pb*NK65 (n = 12) or *Pc*AS (n = 8) relative to naïve mice (dashed line). **(B)** Quantification of 18S parasite DNA transcript in whole brain lysates of *Pb*A- and *Pb*NK65-infected mice (n = 12-13/group) at day 6 post-infection. **(C)** Frequency and total number of *Plasmodium* GAP50-reactive CD8+ T cells in the brains of mice at day 6 post-infection with *Pb*A (n = 14–22), *Pb*NK65 (n = 14–21), or naïve (n = 12). **(D)** Total number of EphA2+ CD8+ T cells in the brains of mice at day 6 post-infection with *Pb*A (n = 14), *Pb*NK65 (n = 14), or naïve (n = 8). **(E)** Frequency of ephrin-A+ CD8+ T cells in the brains of mice at day 6 post-infection with *Pb*A (n = 13), *Pb*NK65 (n = 13), or naïve (n = 6). **(F)** Total number of CD8+T cells and frequency of *Plasmodium* GAP50-reactive CD8+ T cells in the brains of *EphA2-/-* and littermate control mice at day 6 post-infection with *Pb*NK65 (n = 9/group) compared to naïve mice (N) (n = 4/group). Naïve and *Pb*NK65-infected groups are significantly different within each genotype for all graphs except *EphA2+/+* in left panel. **(G)** Transcription of inflammatory chemokines in whole brain lysates of *EphA2-/-* and littermate control mice at day 6 post-infection with *Pb*NK65 (n = 7-10/group) relative to naïve mice (dashed lines). Bars in all graphs represent the mean ± SEM. Statistical analyses: Kruskal-Wallis and Dunn’s multiple comparisons tests (A-left panel, C, D, E, F) and Mann-Whitney test (A-right panel, B, G). Only statistically significant (p<0.05) values are shown unless otherwise noted in the legend. Figures represent combined data from 2 (E, F, G), 3 (A, B, D), or 4 (C) independent experiments.

In support of this hypothesis, upregulation of *EphA2* was not observed in brains of C57BL/6J mice infected with *Pb*NK65 or another *Plasmodium* strain, *Plasmodium chabaudi* AS (*Pc*AS), neither of which causes ECM (**Fig 5A, left**). Significant upregulation only occurs in the brains of mice infected with the ECM-causing strain *Pb*A **(Fig 1A)**. We found no upregulation of ephrin-A1 ligand in the brains of mice infected with either *Pb*A or *Pb*NK65 **(Fig 5A, right)** suggesting that this is not a factor required for the development of ECM. *Pb*NK65 infection of C57BL/6J mice led to equivalent levels of *Plasmodium* transcript **(Fig 5B)** and accumulation of *Plasmodium*-reactive CD8+ T cells in the brain at day 6 post-infection to those measured in *Pb*A infection (**Fig 5C**) consistent with previous reports[15, 28]. CD8+ T cells found in the brains of *Pb*NK65-infected mice expressed similar levels of surface EphA2 (**Fig 5D**) and ephrin-A1 ligand (**Fig 5E**) to those that accumulated in brains of *Pb*A-infected C57BL/6J mice at the onset of ECM. However, unlike *Pb*A-induced CD8+ T cell accumulation in the brain which we found was dependent on EphA2 (**Fig 1E**), *Plasmodium*-reactive CD8+ T cells were found at equal numbers in the brains of *Pb*NK65-infected *EphA2-/-* mice compared to *EphA2+/+* control mice (**Fig 5F**). This is likely due to the fact that levels of chemokines responsible for CD8+ T cell recruitment to the brain are present at equivalent levels in the brains of *Pb*NK65-infected *EphA2+/+* and *EphA2-/-* mice **(Fig 5G)** suggesting that CD8+ T cells are recruited to the brain independently of EphA2 in this non-ECM malaria model.

There are considerable challenges in obtaining brain sections from children who have died from CM as well as control brain sections precluding confirmation that endothelial-expressed EphA2 is the main correlate of BBB breakdown in CM. However, these data from mouse models of *Plasmodium* infection suggest that EphA2 expression on endothelial cells, which is mediated by the interactions of sequestered pRBCs with MBMECs, is a critical mediator of ECM pathogenesis. While expression of *ephrin-A1* ligand in whole brains **(Fig 5A)** and CD8+ T cells **(Fig 5C)** along with soluble ephrin-A1 ligand in the plasma **(Fig 4D)** are similar in *Pb*A and *Pb*NK65 infections, the differential requirement of EphA2 for CD8+ T cell accumulation in the brain in these two mouse models of malaria suggests that the unique and significant difference in brain EphA2 expression may be a contributing factor to the different neurological damage that occurs in these two *Plasmodium* models.

### EphA2 deficiency leads to a reduced neuroinflammatory response to *Pb*A

**Fig 6:**
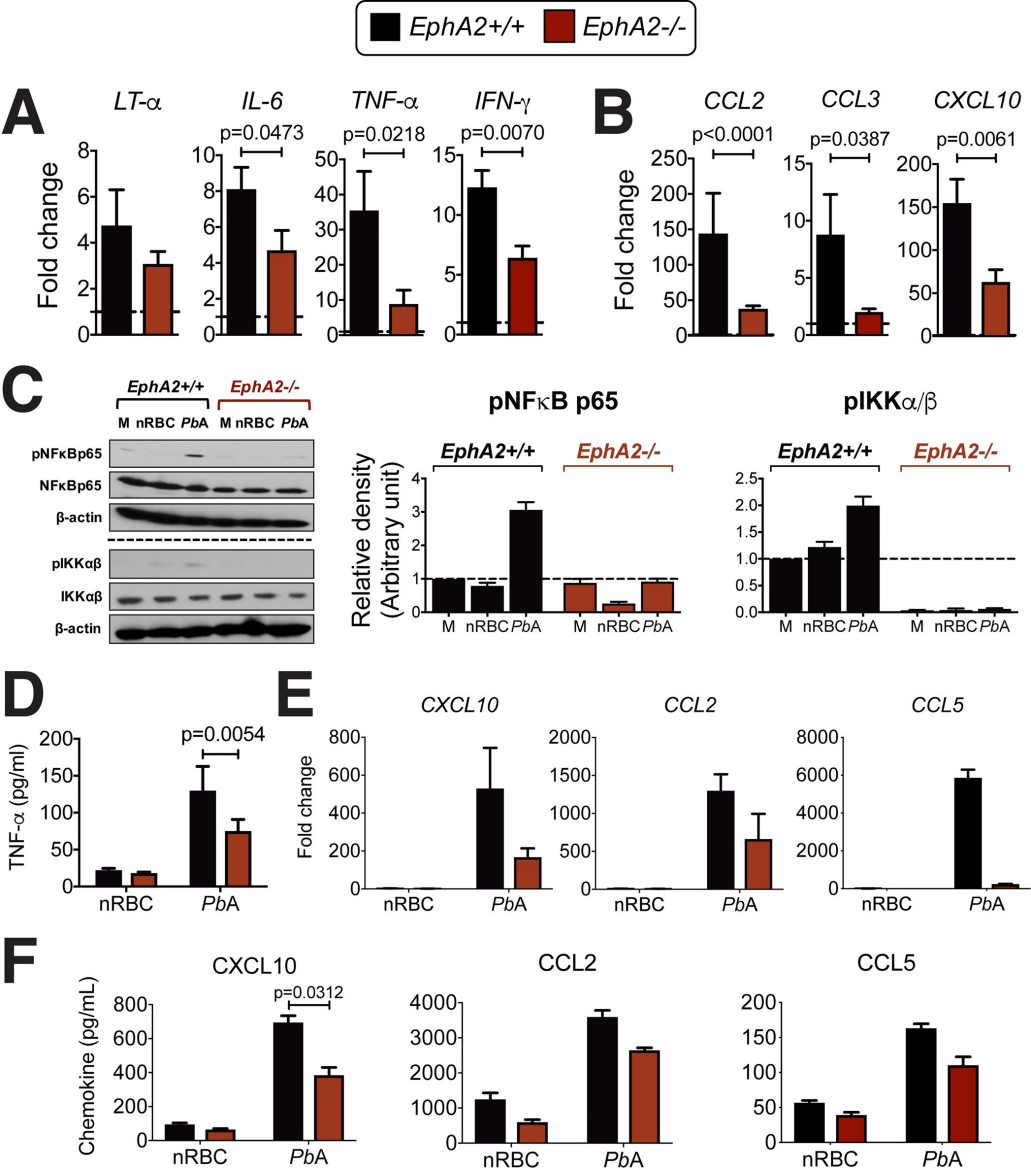
Brain inflammatory response is reduced in *Pb*A-infected *EphA2-/-* mice. Transcription of inflammatory cytokines **(A)** and chemokines **(B)** in whole brain lysates of *EphA2-/-* and littermate control mice at day 6 post-infection with *Pb*A (n = 11-13/group) relative to naïve mice (dashed lines). **(C)** Western blots and densitometry quantification of MBMECs derived from *EphA2-/-* or littermate control mice and incubated with media (M), naïve red blood cell lysate (nRBC), or *Pb*A-infected red blood cell lysate (*Pb*A) for 30 minutes. Blots are representative of 3 endothelial preparations. The horizontal dashed line indicates two separate Western blots. **(D)** TNF-α secreted from identical culture conditions as described in C after 24 hours incubation (n = 4 endothelial preparations). **(E)** Mouse primary brain endothelial cells derived from *EphA2-/-* or littermate control mice were incubated with naïve red blood cell (nRBC) or *Pb*A-infected red blood cell lysate (*Pb*A) for 24 hours and the fold change in the transcription of chemokines relative to unstimulated controls is shown (n = 2 endothelial cultures/group). Chemokines secreted from identical culture conditions are shown in **(F)** (n = 4–6 endothelial preparations/group). Bars in all graphs represent the mean ± SEM. Only statistically significant (p<0.05) values are shown. Statistical analyses: Mann-Whitney test (A-B) and Wilcoxon matched-pairs test (D-F). Only statistically significant (p<0.05) values are shown. Figures represent combined data from 2 (E), 3 (A-C), 4 (D, F-middle and right panels), or 6 (F-left panel) independent experiments.

To determine why CD8+ T cells did not accumulate in the brains of *Pb*A-infected *EphA2-/-* mice despite their abundance in the bloodstream, we examined if there was a defect in the inflammatory response in *EphA2-/-* mice compared to *EphA2+/+* littermate control mice. Transcription of inflammatory cytokines associated with *Pb*A pathogenesis was significantly reduced in brains of *EphA2-/-* mice compared to littermate control mice (**Fig 6A**). In contrast to what was observed in the brains of *Pb*NK65-infected *EphA2-/-* mice **(Fig 5G)**, *Pb*A-infected *EphA2-/-* mice exhibited a significant reduction in the mRNA levels of key chemokines responsible for CD8+ T cell recruitment to the brain during ECM (**Fig 6B**). This suggests that *Plasmodium*-reactive CD8+ T cells do not accumulate in the brain microvasculature of *EphA2-/-* mice because the chemokine signals required for their recruitment to the brain are not present at sufficient levels.

*Plasmodium*-infected RBCs are able to induce a potent inflammatory response in endothelial cells *in vitro*[52]. This includes inducing the production of C-X-C motif chemokine 10 (CXCL10/IP-10)[53, 54], C-C motif chemokine ligand 2 (CCL2/MCP1)[55], and C-C motif chemokine ligand 5 (CCL5/RANTES)[56] which are essential for drawing leukocytes into brain capillaries where pRBCs have adhered to the endothelium. Since EphA2 has been implicated in activation of the NF-κB pathway[57], a critical signaling cascade that mediates inflammation, we investigated if *EphA2-/-* derived MBMECs had reduced NF-κB signaling and inflammatory responses. Stimulation of *EphA2+/+* MBMECs with *Pb*A pRBCs induced phosphorylation of NFκB p65 and IKKα/β, but this effect was abolished in the absence of EphA2 (**Fig 6C**). The reduced phosphorylation of these members of the NF-κB signaling pathway was associated with significantly reduced TNF-α secretion from *EphA2-/-* MBMECs (**Fig 6D**) as well as a clear reduction in transcript levels **(Fig 6E)** and secreted protein **(Fig 6F)** of CXCL10, CCL2 and RANTES in MBMECs stimulated with *Pb*A pRBCs. Together, the reduced cytokine and chemokine production from brain endothelial cells in the absence of EphA2 explain the lack of CD8+ T cells found in the brains of *EphA2-/-* mice during *Pb*A infection and the resulting improvement in survival.

### EphA2 contributes to destabilization of tight and adherens junctions

**Fig 7:**
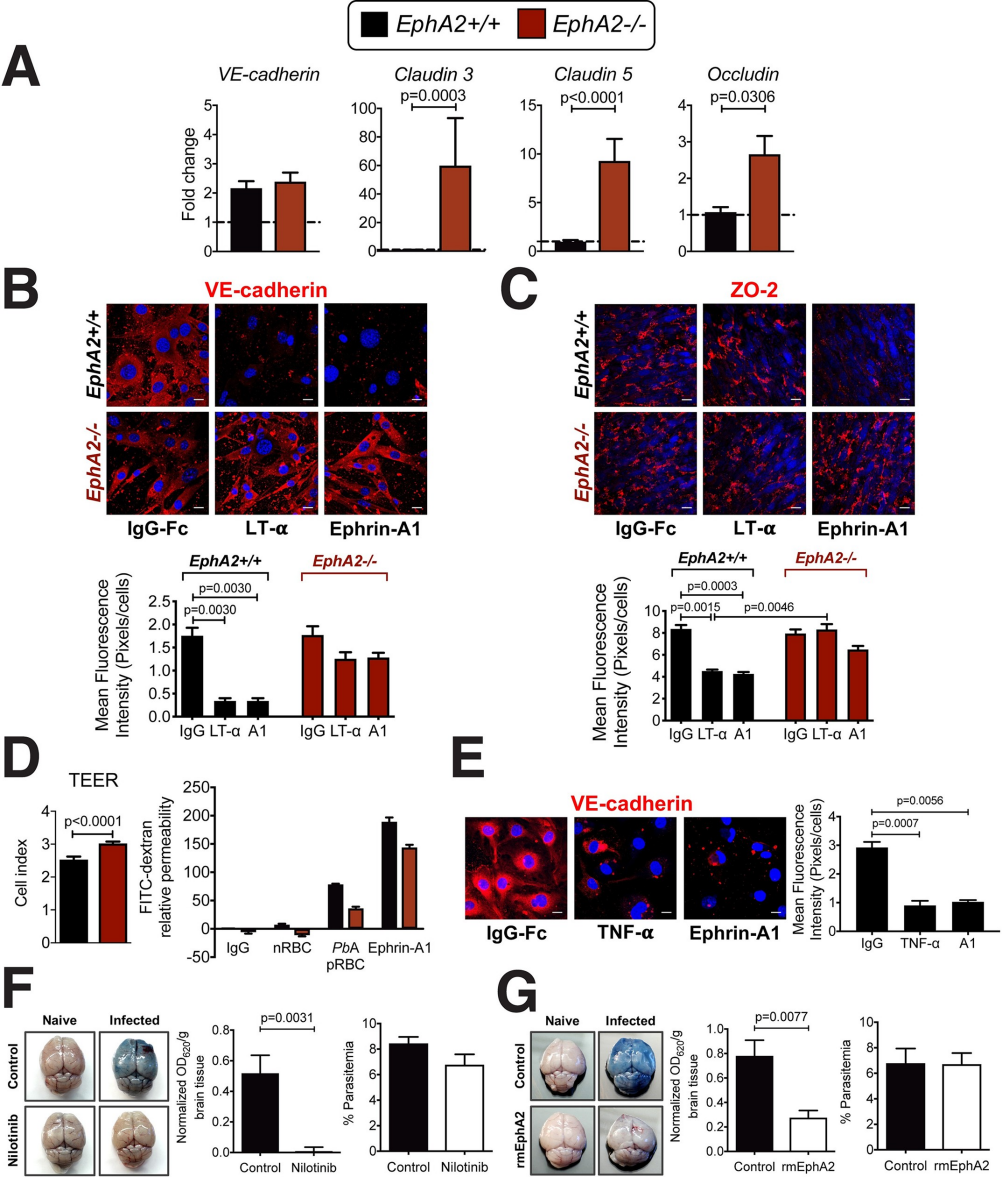
Endothelial cell barrier integrity is enhanced in the absence of EphA2. (**A)** Transcription levels of adherens and tight junction proteins in whole brains of *EphA2-/-* and *EphA2+/+* mice at day 6 post-infection with *Pb*A (n = 8-10/group) relative to naïve mice (dashed line). **(B-C)** Immunofluorescence images and fluorescence quantification of mouse primary brain endothelial cells derived from *EphA2-/-* or littermate control mice showing expression of adherens junction protein vascular endothelial cadherin (VE-cadherin, red) **(B)** and tight junction protein zonula occludens-2 (ZO-2, red) **(C)** after stimulation for 24 hours with recombinant mouse LT-α, recombinant mouse ephrin-A1-Fc, or IgG-Fc as a negative control. Cell nuclei are stained with DAPI (blue). Scale bars represent 25μm. **(D)** Baseline MBMEC transendothelial electrical resistance (TEER, left) and relative permeability (right) of MBMECs from *EphA2-/-* and *EphA2+/+* mice. On right, transwell cultures were incubated for 2 hours with naïve RBC lysate (nRBC), *Pb*A-infected RBC lysate (*Pb*A pRBC), or mouse ephrin-A1-Fc ligand. Permeability is relative to IgG-stimulated *EphA2+/+* endothelial cultures (n = 3 endothelial preparations/group). **(E)** Immunofluorescence images and fluorescence quantification of human primary brain endothelial cells showing expression of VE-cadherin (red) after stimulation for 24 hours with recombinant human TNF-α, recombinant human ephrin-A1-Fc, or IgG-Fc as a negative control. Cell nuclei are stained with DAPI (blue). Scale bars represent 10μm. **(F-G)** Brain permeability in C57BL/6J mice either orally gavaged with 100μL Nilotinib or vehicle control (100 mg/kg/day; n = 8-10/group) **(F)** or injected intraperitoneally with 200μL recombinant EphA2-Fc or vehicle control on days 4–6 post-infection (13.3 μg/mouse/day; n = 4-12/group) **(G)**. Mice were injected intravenously with 200μL of 1% Evan’s Blue at day 6 post-infection with *Pb*A. OD values are normalized to naïve mice from each respective treatment group. Bars in all graphs represent the mean ± SEM. Statistical analyses: Mann-Whitney test (A, D—left, F, G) and Kruskal-Wallis and Dunn’s multiple comparisons tests (B, C, E). Only statistically significant (p<0.05) values are shown. All figures are representative of 2 (A, B, C, E-G) or 3 (D) independent experiments.

Since EphA2 was found to be involved in CD8+ T cell retention in the brain during ECM along with BBB breakdown, we next examined the mechanism by which EphA2 could be contributing to BBB destabilization. In comparison to *EphA2-/-* mice, *EphA2+/+* littermate control mice infected with *Pb*A had significantly reduced transcription of several tight junction proteins (**Fig 7A**) but not the adherens junction protein vascular endothelial cadherin (VE-cadherin). This suggests that the maintenance of an intact BBB in *EphA2-/-* mice was the result of preserved tight junction protein expression. A link between EphA2 activation and dysregulation of adherens and tight junctions in the BBB has been previously shown in other homeostatic and disease contexts[25, 58, 59]. Additional immunofluorescence analyses of MBMECs isolated from *EphA2-/-* and *EphA2+/+* mice and stimulated with ephrin-A1 ligand along with the inflammatory cytokine LT-α revealed significantly reduced expression of both adherens junction **(Fig 7B)** and tight junction **(Fig 7C)** proteins in EphA2 sufficient endothelial cells upon stimulation. On the contrary, expression of these junction proteins was fully maintained in stimulated *EphA2-/-* MBMECs. Using a transwell system we found that in the absence of EphA2, baseline MBMEC barrier integrity appeared more robust than in the presence of EphA2 (**Fig 7D, left**) presumably as a result of enhanced adherens and tight junction protein expression. Activation of EphA2 on MBMECs with ephrin-A1 ligand resulted in the disruption of endothelial barrier integrity (**Fig 7D, right**) with a trend towards decreased permeability in *EphA2-/-* MBMEC cultures. The permeability observed in *EphA2-/-* cultures stimulated with ephrin-A1 ligand could be due to the expression of several other EphA receptors in the brains of *EphA2-/-* mice **(Fig 1F)** that have the potential to bind ephrin-A1 ligand with lower affinity. A similar phenomenon was also observed in HBMECs stimulated with human TNF-α and ephrin-A1 ligand which showed a significant reduction of VE-cadherin expression upon stimulation with both TNF-α and ephrin-A1 ligand (**Fig 7E)**. It is possible that activation of EphA2 upon ephrin-A1 binding leads to either internalization and recycling of junction proteins or EphA2-induced ADAM- and MMP-mediated shedding of junction proteins **(Fig 4D and S5A and S5B Fig**). However, plasma levels of the adherens junction proteins VE-cadherin and epithelial cadherin (E-cadherin) are equivalent or lower in *Pb*A-infected mice compared to naïve mice **(S5C Fig)**. These findings favor the hypothesis that destabilization of junctions occurs through protein internalization either as a direct or indirect (e.g. via suppression of RhoA[25]) result of EphA2 signaling.

### Blocking of the EphA2/ephrin-A ligand pathway confers protection against BBB breakdown

Having shown a pivotal role for EphA2 in mediating BBB disruption during ECM, we assessed whether EphA2 could serve as a novel therapeutic target in an adjunctive therapy for ECM. Treatment of *Pb*A-infected C57BL/6J mice beginning at day 4 post-infection with the receptor tyrosine kinase inhibitor Nilotinib, which has been shown to inhibit multiple receptor tyrosine kinases including EphA2[60] and can cross the BBB unlike related compounds[61, 62], significantly prevented BBB breakdown in comparison to vehicle control treated mice with no effect on peripheral parasitemia **(Fig 7F)**. Given that Nilotinib is not an EphA2-specific inhibitor, we next utilized a strategy aimed at preventing activation of EphA2 through interfering with ephrin-A ligand binding. We treated mice beginning at day 4 post-infection with a recombinant EphA2-Fc protein which has the potential to act as a decoy receptor through binding soluble and cell-bound ephrin-A ligands and preventing their binding to cell-bound EphA2. This treatment also resulted in significantly improved BBB integrity during *Pb*A infection compared to vehicle control treated mice (**Fig 7G)**. These results are notable given that the treatments were only administered beginning at day 4 post-infection, rather than prophylactically, and were still able to prevent the typical onset of BBB breakdown at day 6 post-infection. While neither of these strategies is entirely specific to inhibiting or blocking EphA2 as Nilotinib can act on other receptor tyrosine kinases and recombinant EphA2-Fc treatment will also prevent ephrin ligands from binding to other EphA receptors, these data demonstrate that blocking EphA2 signaling or interaction with its cognate ephrin ligands is a feasible strategy for preventing BBB breakdown during malaria. Given the involvement of Eph receptors and EphA2 in particular in other highly prevalent diseases including several cancers, more targeted therapeutics aimed at blocking binding to and inhibiting activation of EphA2 in a highly specific manner are being developed[63–65]. These are avenues that should also be investigated further in the context of ECM adjunctive therapy.

## Discussion

**Fig 8:**
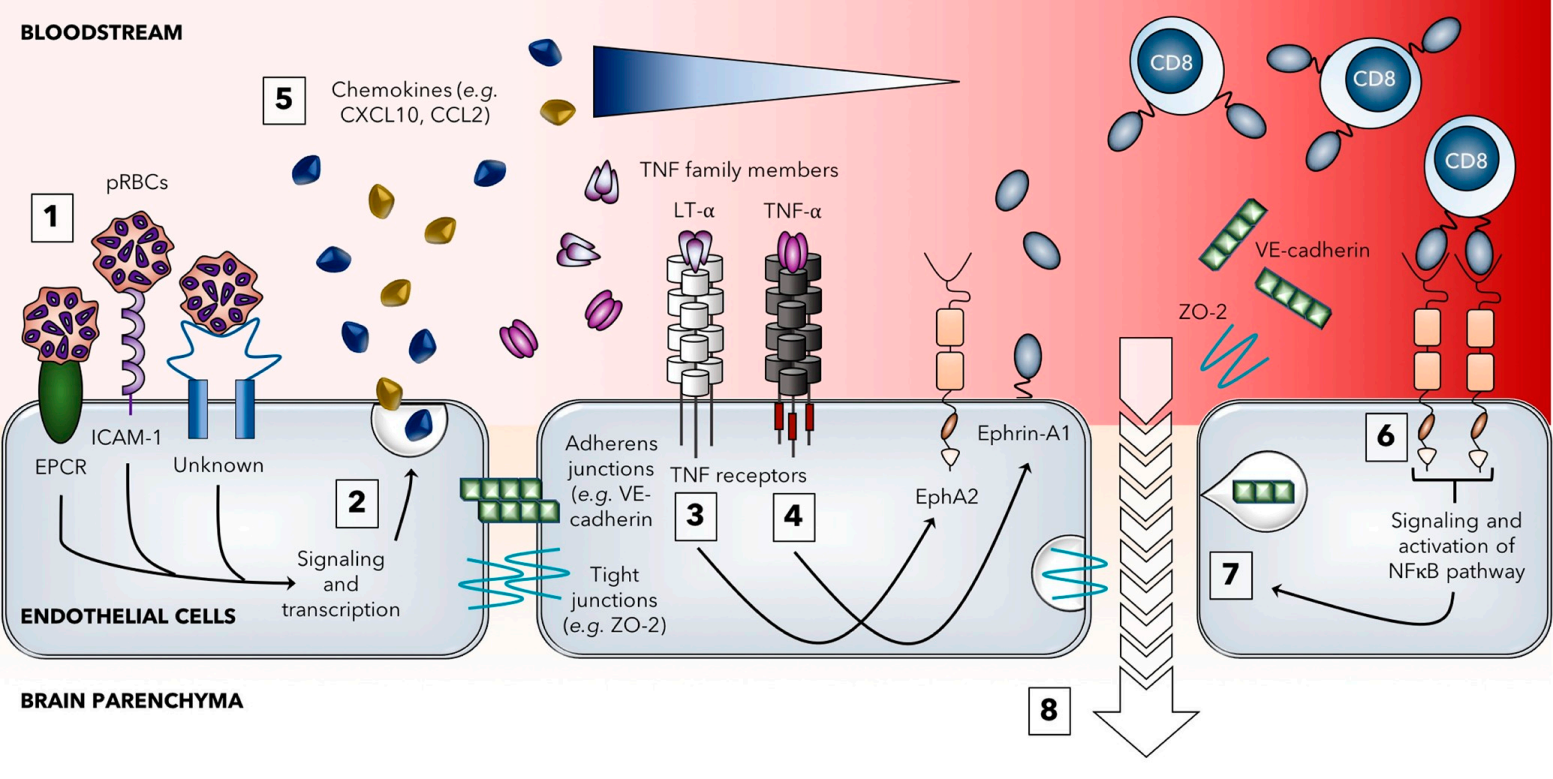
Model for the role of EphA2 in the development of experimental cerebral malaria. The breakdown of the blood-brain barrier during blood-stage *Pb*A infection begins with parasitized red blood cells (pRBCs) in the schizont stage traveling through the bloodstream and adhering to various receptors expressed on brain microvascular endothelial cells including EPCR, ICAM-1, and other unknown receptors that have yet to be identified **(1)**. Signaling through these receptors leads to endothelial activation **(2)** and release of various pro-inflammatory cytokines and chemokines. The cytokine LT-α can act on proximal endothelial cells to induce upregulation of the receptor EphA2 **(3)** while TNF-α induces upregulation of ephrin-A1 ligand **(4)** which can be cleaved by metalloproteinases and released into the bloodstream (although this monomeric form is not believed to signal). Chemokines such as CXCL10 and CCL2 recruit circulating immune cells, including CD8+ T cells, to the brain to the site of inflammation **(5)**. Upon entry into the brain microvasculature, CD8+ T cells expressing ephrin-A1 ligand bind to EphA2 expressed on brain endothelial cells leading to clustering and activation of EphA2. Forward signaling cascades from the EphA2 receptor lead to activation of the NFκB pathway **(6)** which results in various downstream consequences including disruption of endothelial cell junctions due to both internalization and shedding of different adherens and tight junction protein components **(7)**. Once brain endothelial cell junctions are disrupted, contents of the vasculature can leak into the brain parenchyma **(8)** leading to vascular leakage, brain edema, and the development of other neurological symptoms associated with *Pb*A infection. In the absence of EphA2 upregulation or activation (*i*.*e*. EphA2 deficiency, PbNK65 infection, therapeutic targeting), this endothelial junction disruption does not occur and the blood-brain barrier remains intact.

Here we demonstrate that EphA2 is a molecule that mediates BBB breakdown during ECM. Although we have previously shown a critical role for the related Eph receptor, EphB2, in the development of malaria-associated liver fibrosis[66], this study constitutes the first time that any member of this family of Eph receptors has been linked to neurological manifestations of malaria. A critical role for CD8+ T cells in increasing vascular permeability of the BBB in ECM has been repeatedly shown using the *Pb*A mouse model. However, the hypothesis that this occurs predominantly via induction of apoptosis in peptide-MHC I-expressing endothelial cells has little support given the distinct absence of any significant apoptosis in the brain during ECM[15, 17, 30, 67]. Contrary to a pro-apoptotic role for CD8+ T cells and their lytic proteins, our hypotheses that EphA2 upregulation during *Pb*A infection contributes to adherens and tight junction protein dysfunction resulting in increased BBB permeability and that ECM development involves the interaction between EphA2 and CD8+ T cells **(Fig 8)** are consistent with the results of this study.

Indeed, EphA2 has been shown in a variety of contexts to coordinate with and regulate various junction proteins. Through interactions with cadherins[68], EphA2 upregulation and activation appears to destabilize cell-cell contacts[25]. Similarly in the case of tight junction proteins, EphA2 plays a role in the dissolution of tight junctions between endothelial cells which can be prevented through EphA2 inactivation or silencing[26, 69, 70]. It is not clear whether brain endothelial cells lacking EphA2 have higher cellular expression of junction proteins or if *EphA2-/-* mice have a greater density of endothelial cells in the brain. However, our data demonstrates that these EphA2 deficient endothelial cells maintain robust expression of numerous components of tight and adherens junctions which results in a preserved BBB and enhanced survival at a time when *EphA2+/+* mice experience rapid BBB permeability and death. While our data support a critical role for EphA2 expression on brain endothelial cells in mediating BBB breakdown and ECM development, EphA2 is also expressed on CD31 negative cell types known to contribute to BBB maintenance, including pericytes, astrocytes, and neurons[59, 71–74]. As such, future studies should aim to determine the contribution of EphA2 expression on other cellular components of the neurovascular unit to *Plasmodium*-induced BBB junction dysfunction as data on the contribution of these cell types to ECM development is currently very limited[75, 76].

Of particular interest to us are the key differences we observed in EphA2 involvement between *Pb*A infection, which causes ECM, and *Pb*NK65 infection, which does not cause BBB breakdown or cerebral pathology[77, 78]. While these strains are highly conserved, there is not a clear understanding of why infection with *Pb*A, but not *Pb*NK65, leads to ECM development although there is evidence suggesting the existence of differential responses of endothelial cells to the parasites[28]. Given that CD8+ T cells found in the brains of mice infected with *Pb*A and *Pb*NK65 appear to be comparable in terms of their activation status and expression of effector molecules[15], it seems likely that the stark differences in cerebral pathogenesis observed between these two models involve their differential effects on the brain microvasculature. Our finding that EphA2 is uniquely upregulated in brains of mice infected with *Pb*A and not *Pb*NK65 or another non-ECM causing strain (*Pc*AS) highlights EphA2 expression in the brain as a critical correlate of BBB breakdown. Although ephrin-A1 ligand is upregulated on CD8+ T cells and released into the circulation during both *Pb*A and *Pb*NK65 infections, this occurred to the same extent in both infections. Further, the lack of ephrin-A1 ligand upregulation in the brain in both models points to the importance of brain EphA2, not ephrin-A1, in inducing BBB breakdown and ECM development.

Therapeutic options for CM are currently limited to anti-parasitic drugs and supportive care. Our observation that children suffering from neurological manifestations of *P*. *falciparum* infection have elevated levels of ephrin-A1 ligand in the bloodstream demonstrates clinical relevance of our findings. Future studies will aim to determine if EphA2 modulation is present in brains of individuals who succumb to CM as data from our mouse model suggests that this would likely be the primary manner by which Eph receptors could contribute to the BBB dysfunction that has been identified in patients with CM[2–5]. CD8+ T cells along with other cells known to express ephrin ligands including platelets[79] and leukocytes[32] have been found in post-mortem brain histology sections of humans who died of CM[80–84]. Given the known expression of EphA2 in human brain tissue[85] and mounting evidence that endothelial-expressed EphA2 is involved in brain endothelial junction disruption[26, 59], it is quite possible that interactions between EphA2 upregulated on human brain endothelial cells and ephrin-A ligands on other hematopoietic or non-hematopoietic cells could contribute to CM-associated BBB disruption in humans. Although more studies will be needed to elucidate the possible involvement of EphA2 in human CM as well as to determine the optimal method for EphA2 blocking/inactivation, a therapeutic strategy aimed at preventing EphA2-associated junction dysregulation at the BBB could have translational potential.

EphA2 has also recently been shown to serve as a hepatocyte entry receptor for *Plasmodium* sporozoites[86]. While the necessity of this function is not entirely known[87], it further supports the involvement of the EphA2 receptor in *Plasmodium* pathogenesis. Along with the data presented in this study, it suggests that an approach aimed at blocking/inactivating EphA2 could have significant implications for targeting both liver-stage and blood-stage infections. Therapeutic strategies targeting Eph receptors and their ephrin ligands, such as development of antagonistic blocking peptides, are continuously being developed for a number of highly prevalent diseases[88]. Given the growing appreciation for the involvement of Eph receptors, including EphA2, in a variety of infectious diseases[89–91], a better understanding of the optimal strategies for specifically inhibiting a particular Eph receptor while minimizing off-target effects would have broad implications for a number of infectious and non-infectious diseases.

## Materials and methods

### Ethics statement

Blood from healthy controls was obtained from volunteers at Emory University under approval from the Emory University Institutional Review Board (Protocol number 00045690). Ethical approval for the study in Cameroon was obtained from the Emory University (Protocol number 00076693) and University of Utah (Protocol Number 00098806) Institutional Review Boards and from the Cameroon National Ethics Committee (Protocol number 2015/08/622/CE/CNERSH/SP). Administrative authorization of the study was obtained from the Cameroon Ministry of Public Health (Number 6310716). Written informed consents were obtained from the parents or legal guardians of all participants.

### Study population

This study was a cross-sectional study involving 175 children between the ages of 6 months and 17 years of age who presented at the Emergency Units or Outpatient departments of 3 district hospitals (Obala, Efoulan, Nkoleton) and 1 pediatrics reference hospital (Chantal Biya Foundation Hospital) in the Central Region of Cameroon between 2015–2017. The region has continuous transmission of *Plasmodium* peaking between March-May and August-October. The majority of infection (>96%) is caused by *Plasmodium falciparum*. Exclusion criteria included evidence or history of meningitis or encephalitis or a history of developmental delay or other neurological conditions. Individuals were initially tested for *Plasmodium* infection using *Plasmodium falciparum* histidine rich protein II (*Pf*HRPII) and pan-*Plasmodium* species-based rapid diagnostic tests (SD Bioline, S. Korea) followed by examination of thick and thin blood smears to confirm the presence of *Plasmodium*-infected red blood cells. Neurological complications were defined as patients admitted with altered mental status, convulsions, prostration or coma and with visible *Plasmodium*-pRBCs on thick blood smears. Uncomplicated malaria was defined as presence of fever, or history of fever in the last 48 hours with visible *Plasmodium*-pRBCs on thick blood smears with no other signs of severe malaria such as severe malarial anemia (ie. hemoglobin <5g/dL), acidotic breathing or loss of consciousness. Subjects with no visible parasites on thick smears and negative for *Plasmodium* infection by rapid diagnostic test were considered as uninfected endemic controls. All children received the standard clinical care as outpatients or inpatients as applicable. Venipuncture was performed into citrate saline tubes (Vacutainer CPT, BD Biosciences, San Jose, California) and blood samples were refrigerated and transported to the lab and processed within 24 hours. Plasma was frozen at -80°C until used.

### Rodent *Plasmodium* infection

Female C57BL/6J wild type (WT) mice aged 6–12 weeks were bred in-house or purchased from The Jackson Laboratory (Jax stock #000664) (Bar Harbor, ME, USA). *EphA2-/-* whole-body knockout mice on a mixed background (The Jackson Laboratory; JAX stock #006028) were re-derived and bred in-house under a heterozygous breeding system. *TNFRp75-/-* whole-body knockout mice (JAX stock #002620) and *LT-α-/-* whole-body knockout mice (JAX stock #002258) were obtained and used directly for experiments. All mice were given water and food (LabDiet, MO, USA: chow 5001) *ad libitum* and housed under standard conditions. Infections were initiated intraperitoneally with 0.5-1x10^6^ *Pb*A pRBCs (clone15cy1 or reporter line1037cl WT-GFP-Luc^schiz^ mutant RMgm-32[92]), 0.5-1x10^6^ *Pb*NK65 pRBCs (New York Clone), or 1x10^5^ *Pc*AS pRBCs obtained from donor C57BL/6J mice. Peripheral parasitemia was monitored by counting 300–500 RBCs on Diff-Quik (Siemens) stained thin blood smears. Mice were monitored at a minimum of two times a day during experimentation. Parasite burden in tissues was determined by quantitative PCR or using a Xenogen IVIS 100 Bioluminescent Imager after injecting mice with 100μL RediJect D-Luciferin bioluminescent substrate (Perkin Elmer) 30 minutes prior to euthanization and organ dissection for subsequent bioluminescent imaging of parasites. All experiments were approved and carried out according to protocols approved by the Institutional Animal Care Use Committee at Emory University (Protocol number DAR-2000454-021114BN) and University of Utah (Protocol number 17–01001) and the biosafety committees of Emory University (Protocol number HAD01-2425-11R15-101915) and University of Utah (Protocol number 05–17).

### *In vivo* anti-TNFR2 treatment

C57BL/6J mice were intraperitoneally injected with 20μg of either a purified anti-TNFR2 antibody (unconjugated, clone TR75-32.4, BioLegend) or a purified IgG isotype control antibody (unconjugated, clone HTK888, BioLegend) in a volume of 200μL/mouse (diluted in PBS) on days -1, 2, and 5 post-infected with *Pb*A. Mice were euthanized on day 6 post-infection and brains were prepared for RNA extraction and qPCR analyses as described in detail in below sections.

### *Plasmodium falciparum* parasite culture

*P*. *falciparum* parasite lines 3D7 and W2 were cultured under standard conditions. Cultures were grown at a 2% haematocrit in 75-cm^2^ tissue culture flasks at 37°C in medium consisting of human O+ red blood cells in RPMI-1640 (Invitrogen) supplemented with 10% pooled heat-inactivated human A+ serum, 6.0g/L HEPES, 1.8g/L NaHCO_3_, 1.35mg/L hypoxanthine (All from Sigma-Aldrich). Cultures were gassed with a mixture of 3% CO_2_, 1%O_2_ and 96% N_2_ (Airgas). These parasites lines were routinely synchronized by treatment with 5% D-sorbitol (Sigma-Aldrich) and schizont-stage parasites were enriched by centrifugation over a 60% Percoll (Sigma-Aldrich) gradient at 500g for 10min to a purity of ≥95%.

### Isolation of *Pb*A schizonts

*Pb*A-infected mice were euthanized at day 6 post-infection and blood from five infected mice (~2.5mL total) was collected, pooled, and washed extensively with RPMI-1640. The infected RBCs were then transferred to two 75-cm^2^ tissue culture flasks and cultured for 20 hours at a 2% haematocrit in a medium consisting of RPMI-1640, 25mM HEPES, 100μg/ml penicillin, 10U/ml streptomycin, 2mM L-glutamine, 1x sodium pyruvate (all from Sigma) and 10% FCS (Gibco). Cultures were gassed with a mixture of 90%N_2_, 5%O_2_, and 5%CO_2_ (Airgas) prior to incubation at 37°C. *Pb*A schizont-stage parasites were enriched by centrifugation over a 68% Percoll (Sigma-Aldrich) gradient at 450g for 15min. Lysates of pRBCs were made by suspending pRBCs or naïve red blood cells in culture medium at 1x10^7^cells/mL followed by two cycles of freezing and thawing before use.

### PBMC isolation and stimulation

Peripheral blood mononuclear cells (PBMCs) from the blood of malaria-naïve healthy volunteers were isolated by density centrifugation through a Histopaque-1077 gradient (Sigma-Aldrich) and suspended in RPMI-1640 medium supplemented with 10% FCS (Gibco), 1mM sodium pyruvate, 1x penicillin/streptomycin, 2mM L-glutamine and 2-mercaptoethanol (Invitrogen). PBMCs were plated in a 24-well plate (Nunc) at 5x10^5^ per well and incubated overnight in a 5% CO_2_ incubator set at 37°C. The following day, the isolated PBMCs were exposed to *P*. *falciparum* (W2)-enriched schizonts for 48 hours at different ratios. Naïve RBCs were added as a negative control at a ratio of 40:1. In some experiments, isolated PBMCs were depleted of T cells using human CD3 microbeads following the manufacturer’s instructions (Miltenyi) after being exposed to *P*. *falciparum* (3D7) schizont-stage parasites. Stimulated PBMCs were stored in RNA Stat60 (Tel-Test Inc) at -20°C until use.

### *In vivo* Evans Blue blood-brain barrier permeability assay

Mice were injected intravenously with 200μL of 1% Evans Blue (Sigma) on day 6 post-infection and euthanized 1 hour after Evans Blue administration. Brains were removed and imaged using a MicroCapture digital microscope (Veho) and placed into vials containing 1mL formamide (Sigma) at 37°C for 4 days to allow for extraction of Evans Blue dye from whole brain tissue. The concentration of the extracted dye was then measured at an absorbance of 620nm using a plate reader (Biotek). For the treatment experiments, Nilotinib (Selleckchem) was dissolved in DMSO and recombinant mouse EphA2-Fc (rmEphA2) (R&D Systems) was dissolved in PBS. Nilotinib was diluted in water and provided to mice via oral gavage at a dose of 100mg/kg/day in 100μL on days 4–6 post-infection and control mice were given DMSO + water via oral gavage. Naïve control mice were also given Nilotinib and the vehicle control. rmEphA2 was diluted in PBS and intravenously injected into mice at a dose of 13.3μg/mouse/day in 200μL on days 4–6 post-infection and control mice were given PBS intravenously. Naïve control mice were also given rmEphA2 and the vehicle control.

### Primary human brain microvascular endothelial cells

Primary human brain microvascular endothelial cells (HBMECs) used in this study were either gifted or obtained from a commercial source (Creative Dynamics Inc). HBMECs were cultured in SuperCult endothelial cell growth medium following the supplier instructions (Creative Dynamics Inc). Endothelial cells (HBMEC) were used between passage 2 and 5.

### Isolation and culture of primary murine brain microvascular endothelial cells

The technique for isolating primary murine brain microvascular endothelial cells (MBMECs) has been described elsewhere[93]. Briefly, at least 5 mice (8–12 weeks old C57BL/6J or *EphA2+/+* and *EphA2-/-*) were euthanized, the brain extracted, and the brain stems, cerebella, and thalami removed and discarded while the cerebra were transferred into a falcon tube containing DMEM-F12 medium (Gibco). The tissue was minced by pipetting up and down several times and digested with a mixture of collagenase D (10mg/mL) and DNAse (1mg/mL) (Roche) in DMEM-F12 medium for 1 hour at 37°C on an orbital shaker at 180rpm. DMEM-F12 (10mL) was added to the tissue suspension and the homogenate centrifuged at 1000g for 10 min at 4°C. De-myelination of brain homogenate was achieved by mixing the pellet in 25mL of 20% BSA-DMEM/F12, thoroughly pipetting several times, and centrifuging at 1000g for 20 min. at 4°C. The upper myelin layer was discarded and the pellet containing endothelial cells (ECs) was washed twice with DMEM-F12 supplemented with 1x MEM vitamin, 1x MEM amino acid, 1x antibiotic-antimycotic (Sigma-Aldrich), 1mM sodium pyruvate and 2-mercaptoethanol denoted “incomplete DMEM/F12” (iDMEM/F12). Next, the pellet was resuspended in red blood cell lysis buffer (eBioscience), incubated on ice for 5 min., and centrifuged for 5 min. at 1000g. The cells were then gently overlaid onto a 30% Percoll gradient and centrifuged for 10 minutes at 700g at 4°C with no acceleration or brake. The interface containing endothelial cells was carefully removed and washed with iDMEM/F12 twice and suspended in iDMEM/F12 supplemented with 20% FCS (Gibco), 100μg/mL endothelial cell growth supplement (BD Biosciences), 1 unit of Heparin (Hospira Inc.) (complete endothelial cell medium) and 10μg/mL puromycin (Sigma-Aldrich). Endothelial cells were then plated in a 6-well plate and incubated at 37°C in a 5% CO_2_ incubator. After two days of culture, puromycin was removed from the endothelial cell medium described above and ECs were cultured until they reached 90% confluency. The purity of the endothelial cell preparation was verified by immunofluorescence staining for the endothelial cell marker von Willebrand factor (VWF) and flow cytometry staining with an anti-CD31 antibody. Primary MBMEC were used between passage 2 and 5.

### Stimulation of endothelial cells

Recombinant human and mouse tumor necrosis factor-alpha (TNF-α) and lymphotoxin-α (LT-α) were used at a concentration of 10ng/mL (R&D Systems). EphA2 (Catalog # 639-A2) and ephrin-A1 (Catalog # 602-A1) ectodomain-human IgG1 Fc fusion proteins were from R&D Systems. Purified human IgG-Fc protein was from Calbiochem (EMD Millipore Catalog # 401104). EphA2-Fc, Ephrin-A1-Fc, and IgG proteins were used at a concentration of 5μg/mL and were clustered by mixing with anti-human IgG-Fc in a 2:1 w/w ratio and incubated at 22°C for 1 hour or overnight at 4°C. This treatment allows the soluble Fc proteins to reproduce the clustering that occurs on cell membranes necessary for the initiation of biologically relevant signaling. Endothelial cells were also stimulated with 50μL of either naïve RBC or *Pb*A-infected RBC lysates.

### Brain sectioning

Brain sections prepared for immunofluorescence staining were collected from 5–9 week old *EphA2-/-*, *EphA2+/+*, or C57BL/6J mice at day 6 post-infection with *Pb*A or Krebs saline for naïve control mice. Extracted brains were bisected to separate the left and right hemispheres and incubated in 4% paraformaldehyde (PFA) overnight at 4°C. Afterwards, brains were transferred to a 30% sucrose and 70% of 4% PFA solution and incubated for seven days at 4°C. To embed in preparation for slicing, brains were placed in embedding molds and subsequently submerged in Tissue-Tek O.C.T. compound (Sakura Finetek) before freezing with dry ice. Once frozen, the molds were stored in -80°C for a maximum of two weeks prior to sectioning. 8 micron thick sagittal sections were obtained using a cryostat maintained at -20°C working temperature. Sections were collected on Shandon Colorfrost Plus microscope slides (ThermoScientific) and stored at -20°C until used for immunofluorescence staining.

### Immunofluorescence staining

For staining of endothelial cell cultures, cells were seeded on gelatin-coated chamber slides (Lab-Tek, 8 wells, glass) and grown to confluence before being serum starved for 4 hours in iDMEM/F12 containing 1% BSA prior to stimulation. After stimulation, cells were washed with PBS, fixed with 2% paraformaldehyde (PFA) (Sigma-Aldrich) solution for 30 min. at room temperature, and permeabilized using 0.5% Triton X-100/Tris-HCl (Sigma-Aldrich) (100mM) for 10 min. at room temperature, blocked with 5% donkey serum (Abcam) for 1 hour and incubated overnight at 4°C with the following primary antibodies: rabbit polyclonal to von Willebrand factor (1:500, ab9378, Abcam), rabbit polyclonal to VE-cadherin (1:50; ab33168, Abcam), goat polyclonal to VE-cadherin (1:50, AF1002, R&D Systems), ZO-2 (1:50; Cell Signaling Technology), FITC-conjugated lectin from *Lycopersicon esculentum* (tomato) (1:100, L0401, Sigma), and mouse monoclonal to EphA2 (1:50, 1C11A12, Thermo Fisher). Cells were then stained with NorthernLights 493 or NorthernLights 577 conjugated secondary antibodies for 1 hour (1:500; R&D Systems). Nuclei were counterstained with mounting medium containing DAPI (VectorShield). Brain sections were processed and stained using the same protocol. Images were taken at room temperature using a Nikon A1 confocal microscope with either a 20X (numerical aperture: 0.75) or 60X (numerical aperture: 1.4) objective lens using NIS-Elements acquisition software. ImageJ software (NIH) was used for image processing post-acquisition and at least ten random fields were analysed for quantification.

### Transmission electron microscopy

For TEM examination of endothelial cell cultures, the samples were fixed with 2.5% glutaraldehyde in 0.1 M sodium cacodylate (pH 7.4). Samples were then washed with the same buffer twice and post-fixed with 1% osmium tetroxide and 1.5% potassium ferrocyanide, dehydrated through a graded ethanol series to 100%, and embedded in Eponate 12 resin (Ted Pella Inc., Redding, CA). Ultrathin sections were cut on a Leica UltraCut S ultramicrotome (Leica Microsystems Inc., Buffalo Grove, IL) at 70 nm, and counter-stained with 4% aqueous uranyl acetate and 2% lead citrate. Sections were examined using a 120 kV JEOL JEM-1400 LaB6 transmission electron microscope (JEOL, Ltd., Japan).

### ELISA

Mouse ephrin-A1 (R&D Systems), VE-cadherin (Abcam), and E-cadherin (Abcam) ELISA kits were used to detect the presence of soluble ephrin-A1 ligand, VE-cadherin, and E-cadherin, respectively, in the plasma of mice. Mouse ephrin-A1 ELISA kits were also used to detect soluble ephrin-A1 present in MBMEC culture supernatants post-stimulation as described in detail in a previous section. Mice were euthanized with isoflurane and whole blood was collected into heparinized tubes and centrifuged at 1000g for 15 minutes at 4°C to isolate plasma. Plasma was diluted 1:10 for ELISA analysis of ephrin-A1 ligand, 1:800 for ELISA analysis of VE-cadherin, and 1:2000 for ELISA analysis of E-cadherin. Absorbance was measured at 450nm using a plate reader (Biotek) and plasma concentrations were determined using standard curves as per the manufacturer’s instructions. Human ephrin-A1 (Sino Biological) and human EphA2 (R&D Systems) ELISA kits were used to detect the presence of soluble ephrin-A1 ligand and soluble EphA2, respectively, in the plasma of humans and carried out according to the manufacturer’s instructions.

### Luminex of endothelial cell culture supernatants

Cytokine and chemokine analysis from EC culture supernatant was performed using Singleplex Luminex kits for each analyte tested according to the manufacturer’s instructions (eBioscience and ThermoFisher Scientific).

### *In vitro* endothelial cell permeability assay

Transendothelial electrical resistance (TEER) was measured by Electric Cell-substrate Impedance Sensing (ECIS). An 8W10E+ electrode 96-well culture array (Applied Biophysics) was coated with 0.1% gelatin (Sigma) and 10 μg/mL human fibronectin. 1 x 10^4^ primary MBMECs isolated from *EphA2+/+* and *EphA2-/-* mice per well were seeded in complete media onto the electrode culture array and monitored until a stable monolayer formed. Once a stable measurement of resistance from MBMECs was achieved, resistance changes were further monitored in real time for up to 48 hours. Data were analyzed using the X-CELLigence experiment report software.

To assess paracellular permeability, primary MBMECs from *EphA2+/+* and *EphA2-/-* mice were seeded on gelatin-coated transwell filter plates (Costar 3413, 0.4μm pore size; Corning) and grown to confluence. For stimulation, MBMECs were serum starved for 4 hours in iDMEM/F12 containing 1% BSA, then pre-incubated with the lysates of naïve RBCs and *Pb*A-schizonts (pRBCs), 5μg/mL of cluster-activated ephrin-A1-Fc and EphA2-Fc (for 2 hours) parallel to the diffusion of 25μg/mL FITC-dextran (250kD; Sigma-Aldrich) at 37°C and 5%CO_2_. Fluorescence in the lower chamber was measured with a plate reader (BioTek) at 490nm.

### RNA extraction and cDNA synthesis

Cells or tissue were homogenized in RNA-Stat60 (Tel-Test Inc) and total RNA was extracted using standard phenol-chloroform protocols followed by DNAse treatment of the RNA extracted using RNA-II purification kit (Nachery-Nagel). A total of 100ng of RNA per sample was converted into cDNA using Superscript II (Life Technologies) at 42°C for 50 min., 70°C for 15 min., in the presence of 5uM oligo (dT)_16-18_, 5mM Dithiothreitol (DTT), 0.5mM dNTPs (all from Life Technologies), 8U RNAsin (Promega), 50mM Tris-HCl pH8.3, 75mM KCl and 3mM MgCl_2_. The cDNA was treated with 2.5U RNAse H (Affymetrix) at 37°C for 20min to remove any remaining RNA residues.

### Quantitative PCR

Real-time qPCR reactions were performed using Quantitect SYBR Green PCR reagent (Qiagen). PCR amplification was performed with 5μL cDNA per sample (diluted 1:10), 2μM of each primer, and 7μl of QPCR SYBR green mix. Plates were then run using Applied BioSystems FAST 7000 Sequence detection system (ABI Prism FAST 7000). Mouse primer sequences are shown in S1 Table and human primer sequences are shown in S2 Table. Transcripts were normalized to a housekeeping gene (Ubiquitin or β-actin) and expression levels calculated using the 2^-ΔΔCt^ method[94]. The fold change of transcription for individual infected animals was calculated in relation to the average expression in naïve mice for each group at each time point.

### Tissue processing for flow cytometry

Spleens were pressed through a 40μm cell strainer and suspended in Iscove’s Modified Dulbecco’s Medium (IMDM) containing 100units/mL penicillin, 100μg/mL streptomycin, 1μM L-glutamine, 12mM HEPES, 0.5mM sodium pyruvate, 5 X 10^-5^M 2-mercaptoethanol (all Gibco) (cIMDM). Single cell suspensions of splenocytes were centrifuged at 1500rpm for 8 minutes at 4°C prior to RBC lysis of the pellet using an NH_4_Cl-based RBC lysis buffer (BioLegend). Splenocytes were centrifuged again before resuspension in cIMDM with the addition of 10% heat-inactivated fetal calf serum (FCS) (PAA Laboratories) for downstream flow cytometric analysis. Brains were pressed through a 100μm cell strainer and resuspended in a 30% Percoll solution before overlaying onto a 70% Percoll gradient. Brain samples were centrifuged at 600g for 20 minutes at room temperature with no brake. The peripheral blood mononuclear cell (PBMC) interface was collected and washed with cIMDM + 10% heat-inactivated FCS before resuspension in cIMDM + 10% heat-inactivated FCS for downstream flow cytometric analysis. Lungs were pressed through a 40μm cell strainer, resuspended in cIMDM containing Liberase-TL synthetic collagenase (Roche) at a final concentration of 0.3mg/mL and dispase (Invitrogen) at final concentration 2mg/mL and further incubated for 45 min. at 37°C. The suspension was overlaid on a 30% Percoll gradient and centrifuged at 1800g for 10min. The pellet was collected and RBCs were removed from suspensions by incubation in an NH_4_Cl-based RBC lysis buffer (BioLegend).

For flow cytometry, samples were incubated with Fc-blocking antibodies (anti-mouse CD16/CD32, clone 2.4G2, BioLegend) followed by various combinations of the following antibodies: CD3 (PE-Cy5, clone 145-2C11, BioLegend), CD4 (PerCP-Cy5.5, clone GK1.5, BioLegend), CD8 (BV711, clone 53–6.7, BioLegend), CD44 (Alexa 700, clone IM7, eBioscience) Zombie NIR viability dye (BioLegend), and EphA2 (PE, clone 233720, R&D Systems). For detection of *Plamodium*-specific CD8+ T cells, previously identified tetramers recognizing the glideosome-associated protein 50 epitope 40–48 (GAP50-PE)[28] and the replication protein A1 epitope 199–206 (F4-APC)[29] were generated by the NIH Tetramer Core Facility and incorporated into the flow panels. For detection of surface ephrin-A1 ligand expression, samples were first incubated with 2μg/mL recombinant mouse EphA2-Fc protein (R&D Systems) for 1 hour at room temperature prior to incubation with a secondary anti-human IgG-Fc antibody (PE or FITC, clone HP6017, BioLegend). Intracellular cytokine staining on splenocytes was undertaken after incubation for 6 hours at 37°C in a 96-well plate containing immobilized anti-CD3 (50μL/well at 5μg/mL) (clone 17A2) and soluble CD28 (2μg/mL) (clone 37.51) (eBioscience) in the presence of 10μg/mL brefeldin A (BioLegend). After surface staining, cells were fixed with a paraformaldehyde-based fixation buffer (BioLegend), permeabilized with a permeabilization buffer (BioLegend), and stained with anti-IFN-γ-PE (clone XMG1.2, BioLegend) and anti-Granzyme B-Alexa 647 (clone GB11, BioLegend). Flow cytometric acquisition was performed on BD LSRII and BD LSRFortessa flow cytometers (BD Biosciences) and data analyzed using FlowJo software (TreeStar).

### Fluorescence activated cell sorting of splenocytes

For sorting of splenic T cells, B cells were first removed using CD19+ positive selection beads (Miltenyi Biotech) and populations were sorted from CD19 negative splenocytes. CD4+ T cells (CD3+CD4+CD8-) and CD8+ T cells (CD3+ CD4-CD8+CD11c-) were sorted on a FACS Aria II (BD Biosciences) before being stored in RNA Stat-60 at -80°C until processed for RT-qPCR as described in detail in a previous section.

### Western blot

Endothelial cell lysates were prepared with RIPA buffer containing 1x EDTA/proteinase-phosphatase inhibitor cocktail (Pierce). Protein concentration was determined using a BCA kit (Thermo Scientific). The supernatant lysate was stored at -80°C until used for immunoblotting. Protein extracts were separated by SDS-PAGE electrophoresis and blotted onto nitrocellulose membranes. Blots were incubated overnight with the following primary detection antibodies: anti-mouse pNFκBp65 (clone 93H1), NFκBp65 (clone D14E12), pIKKαβ (clone 16A6) (all from Cell Signalling Technology and used at a 1:1000 dilution), anti-mouse IKKαβ (clone 42D1) at a 1:500 dilution (Pierce), or β-actin (clone AC-15) at a 1:10,000 dilution (Pierce). Blots were then stained for 1 hour with mouse or rabbit HRP-conjugated secondary antibodies used at a 1:2000 dilution (R&D Systems). Finally, blots were developed using ECL substrate per the manufacturer’s instructions (Pierce) and quantified using densitometry measurements on ImageJ software.

### Statistical analyses

Using Prism software, differences between two groups of animals were assessed using a non-parametric 2-tailed Mann Whitney test or a 2-tailed parametric t-test as stated. Paired samples were tested using a non-parametric 2-tailed Wilcoxon matched pairs signed rank test or a paired t-test. Survival analysis was performed using a Log-rank Mantel-Cox test. Comparisons of more than two groups were assessed using non-parametric Kruskal-Wallis test followed by Dunn’s multiple comparisons test. General Linear Modelling (GLM), a variant of analysis of variance (ANOVA) including all 1^st^ order interactions, was performed using Minitab software (Minitab, Inc.) to determine the statistical significance of soluble ephrin-A1 or EphA2 in patient samples taking into account age, area of residence, and peripheral parasitemia levels. Residual variation was assessed for normality using Anderson-Darling test and heterogeneity of variance using the F-test. To meet the requirements of parametric testing, both soluble EphA2 and ephrin-A1 data were logarithmically transformed prior to analysis. F values quoted are from the minimal model of the data with all insignificant terms removed. In all cases, P values are stated in the figures and P<0.05 was considered statistically significant. Only significant differences are shown as stated.

## Supporting information

S1 Fig Parasite sequestration differs in spleen and liver, but not lung or brain, of *EphA2-/-* mice. **(A)** Representative images of *Pb*A schizonts expressing luciferase under the AMA-1 promoter sequestered in brains isolated from *EphA2-/-* and littermate control mice at day 6 post-infection in comparison to brains from naïve mice. **(B)** Quantification and representative images of *Pb*A schizonts expressing luciferase under the AMA-1 promoter (n = 7-8/group) sequestered in spleen, liver, and lung tissue of *EphA2-/-* and littermate control mice at day 6 post-infection. Bioluminescence values are normalized to naïve mice from each respective group. Bars in all graphs represent the mean ± SEM. Statistical analyses: Mann-Whitney test (B). Only statistically significant (p<0.05) values are shown. Figures are representative of 2 (A-B) independent experiments.
(TIF)

S2 Fig EphA2 protein is expressed on brain endothelial cells and upregulated during *Pb*A infection. **(A)** Immunofluorescence images demonstrating co-expression of the lectin-labeled vasculature (green) and EphA2 (red) in the cortex of sagittal slices from brains of *EphA2-/-* and *EphA2+/+* mice isolated at day 6 post-infection with *Pb*A compared to naïve mice. Cell nuclei stained with DAPI (blue). Scale bars represent 25μm. Images representative of 2 independent experiments.
(TIF)

S3 Fig *EphA2* expression is not upregulated in the liver and lung and EphA2 is not required for CD8+ T cell migration to the lung during *Pb*A infection. Transcription of EphA receptors relative to naïve mice (dashed line) in liver (n = 8/group) **(A)** and lung (n = 7-11/group) **(B)** lysates of C57BL/6J mice at day 6 post-infection with *Pb*A. **(C-D)** Frequency (left) and total number (right) of *Plasmodium* GAP50-reactive (n = 8-9/group) **(C)** and *Plasmodium* F4-reactive (n = 8-9/group) **(D)** CD8+ T cells present in the lungs of *EphA2-/-* and littermate control mice at day 6 post-infection with *Pb*A compared to naïve mice (N) (n = 4/group). Naïve and *Pb*A-infected groups are significantly different within each genotype for all graphs. Bars in all graphs represent the mean ± SEM. Statistical analyses: Mann-Whitney tests (C-D). Only statistically significant (p<0.05) values are shown unless otherwise noted in the legend. Figures are representative of 2 (A-D) independent experiments.
(TIF)

S4 Fig Exposure to *Plasmodium* increases transcription of ephrin-A ligands in human PBMCs, particularly CD3+ T cells, and mouse CD4+ and CD8+ T cells. **(A-B)** Transcription of ephrin-A1 and ephrin-A4, ligands known to bind with high affinity to EphA2, in PBMCs isolated from healthy human donors incubated with naïve red blood cell lysates (nRBC) or *P*. *falciparum*-infected red blood cell lysates (*Pf* pRBC) (clone W2) at different ratios for 48 hours. **(C-D)** Transcription of ephrin-A1 and ephrin-A5 ligands in PBMCs isolated from healthy human donors incubated with naïve red blood cells lysates (nRBC) or *P*. *falciparum*-infected red blood cell lysates (pRBC) (clone 3D7) at a ratio of 40:1 for 48 hours before and after CD3+ T cell magnetic depletion. Boxes in A-D represent the median ±25^th^ and 75^th^ percentiles with minimum/maximum whiskers and transcription is relative to unstimulated PBMCs (N). **(E)** Transcription of ephrin-A1 and ephrin-A5 ligands on CD4+ and CD8+ T cells sorted from the spleens of C57BL/6J mice at day 5 post-infection with *Pb*A (n = 8) relative to naïve mice (dashed line). **(F)** Levels of soluble EphA2 in the plasma of children living in an area in Cameroon endemic for *P*. *falciparum* malaria. Patients were categorized by admission to the hospital for neurological complications (n = 51), uncomplicated malaria (n = 50), or uninfected and presenting for routine pediatric tests (n = 49). Each dot represents an individual patient. Bars in E-G represent the mean ± SEM. Statistical analyses: Kruskal-Wallis and Dunn’s multiple comparisons tests (A-D) and General linear modeling and Tukey’s pairwise comparison post-ANOVA (G). Only statistically significant (p<0.05) values are shown. Figures are representative of 2 (E), 4 (A, B), or 6 (C, D) independent experiments.
(TIF)

S5 Fig Transcription of metalloproteinases is upregulated in the spleen and brain during the course of *Pb*A infection. Transcription of a disintegrin and metalloproteinase domain-containing proteins (ADAM-10, ADAM-12, ADAM-17) with thrombospondin motifs (ADAMTS-13) (A) and matrix metalloproteinases (MMP-3, MMP-8, MMP-9, MMP-13) (B) relative to naïve mice (dashed line) in spleen (n = 4/day) and brain (n = 4/day) lysates of C57BL/6J mice at different time points post-infection with *Pb*A. ND indicates no transcript was detected. (C) VE-cadherin (left) and E-cadherin (right) present in the plasma of C57BL/6J mice at day 6 post-infection with *Pb*A (n = 11-14/group) compared to naïve mice (n = 4-6/group). Bars in all graphs represent the mean ± SEM. Statistical analyses: Mann-Whitney test (C). Only statistically significant (p<0.05) values are shown. Figures are representative of 1 (A-B) or 2 (C) independent experiments.
(TIF)

S1 Table Comprehensive list of mouse primers. All mouse forward and reverse primer sequences used for mRNA amplification and RT-qPCR assays for the genes listed, including data found in both primary and supplemental figures, are included in this table.
(DOCX)

S2 Table Comprehensive list of human primers. All human forward and reverse primer sequences used for mRNA amplification and RT-qPCR assays for the genes listed, including data found in both primary and supplemental figures, are included in this table.
(DOCX)
