# Supplementary figures and images for: EphA2 contributes to disruption of the blood-brain barrier in cerebral malaria
Source: PLoS Pathog. 2020 Jan 30;16(1):e1008261. doi: 10.1371/journal.ppat.1008261 (PMC6991964; doi:10.1371/journal.ppat.1008261)

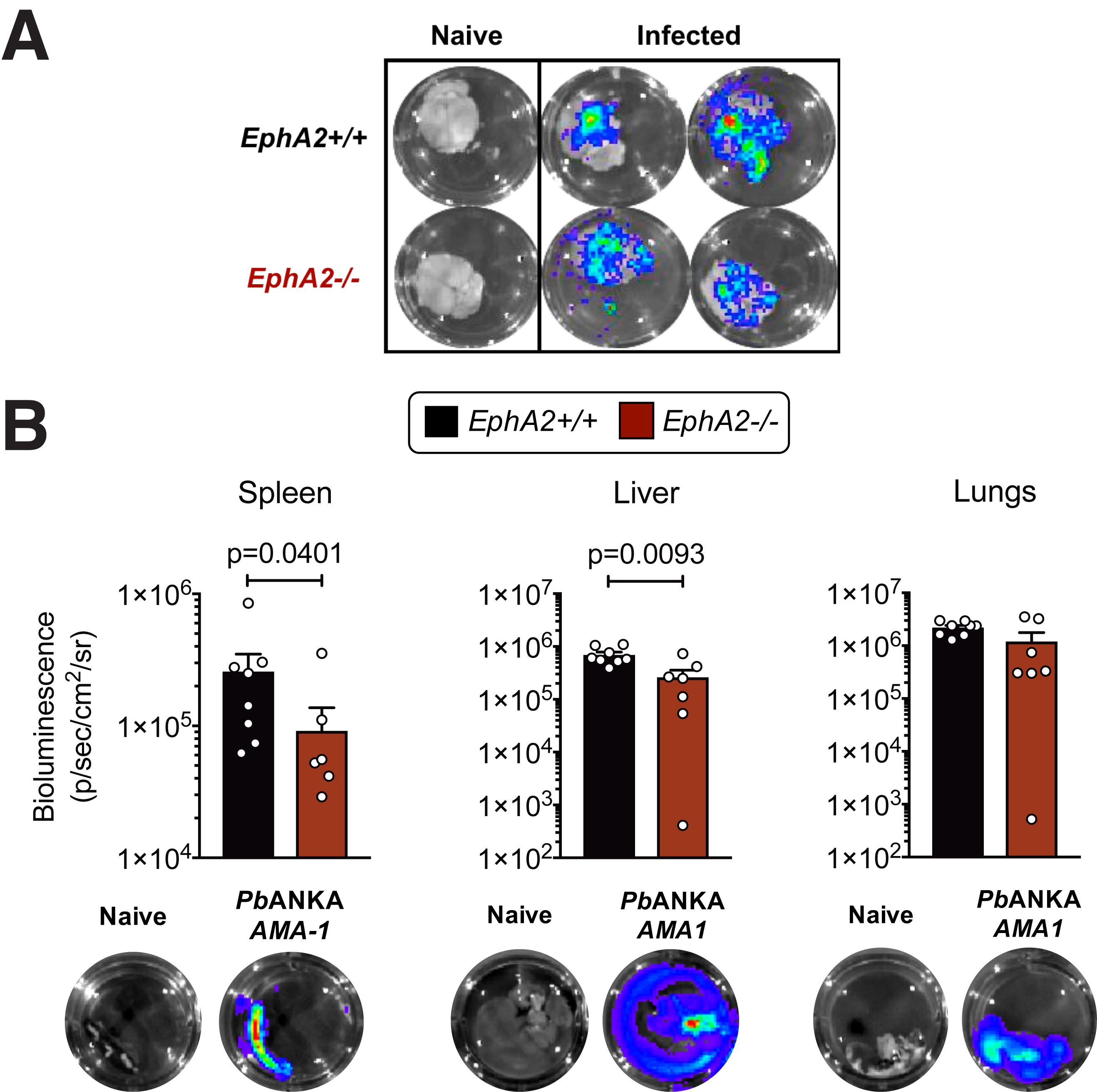

Supplement: S1 Fig — (A) Representative images of PbA schizonts expressing luciferase under the AMA-1 promoter sequestered in brains isolated from EphA2-/- and littermate control mice at day 6 post-infection in comparison to brains from naïve mice. (B) Quantification and representative images of PbA schizonts expressing luciferase under the AMA-1 promoter (n = 7-8/group) sequestered in spleen, liver, and lung tissue of EphA2-/- and littermate control mice at day 6 post-infection. Bioluminescence values are normalized to naïve mice from each respective group. Bars in all graphs represent the mean ± SEM. Statistical analyses: Mann-Whitney test (B). Only statistically significant (p<0.05) values are shown. Figures are representative of 2 (A-B) independent experiments. (TIF) [file ppat.1008261.s001.tif]

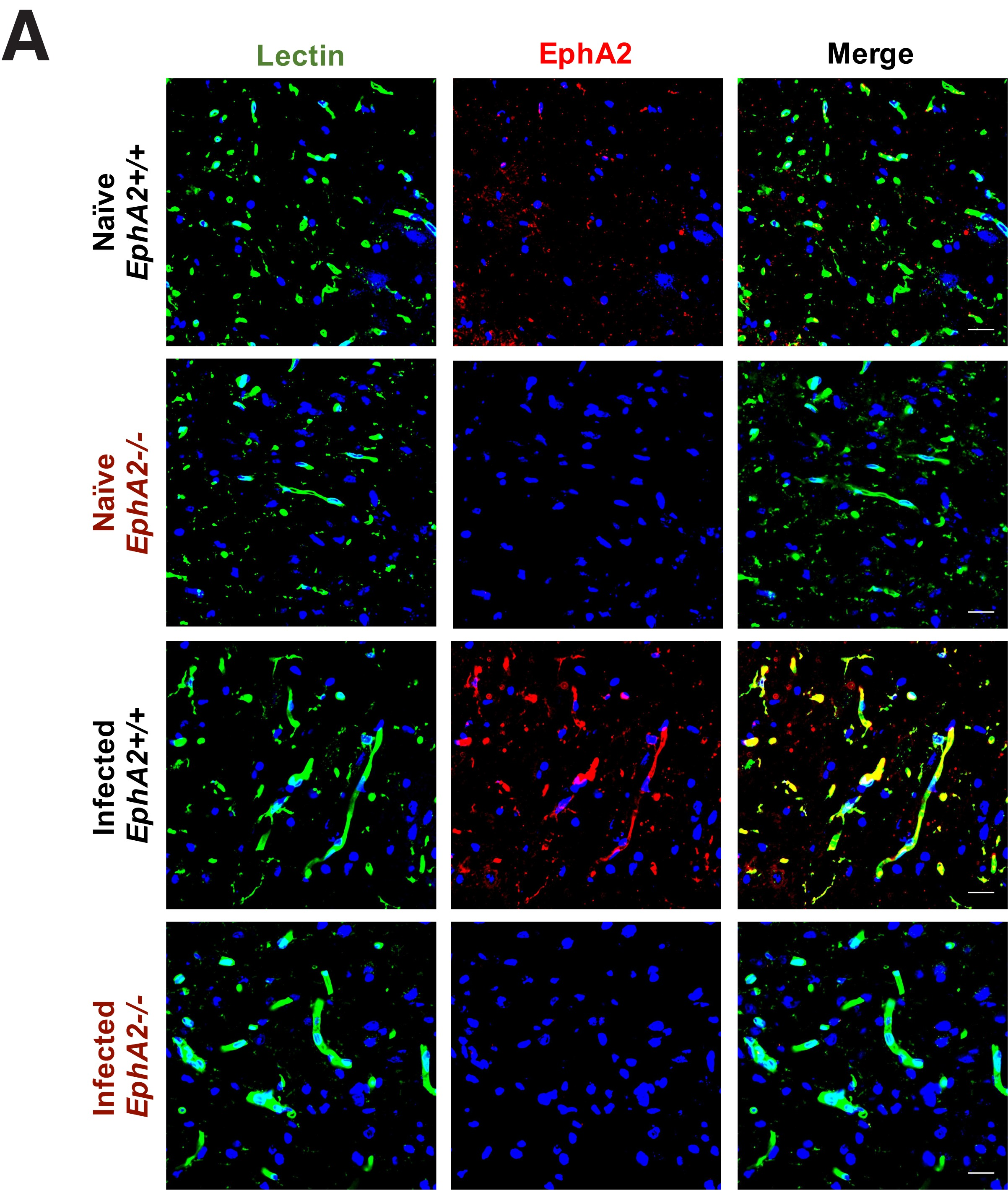

Supplement: S2 Fig — (A) Immunofluorescence images demonstrating co-expression of the lectin-labeled vasculature (green) and EphA2 (red) in the cortex of sagittal slices from brains of EphA2-/- and EphA2+/+ mice isolated at day 6 post-infection with PbA compared to naïve mice. Cell nuclei stained with DAPI (blue). Scale bars represent 25μm. Images representative of 2 independent experiments. (TIF) [file ppat.1008261.s002.tif]

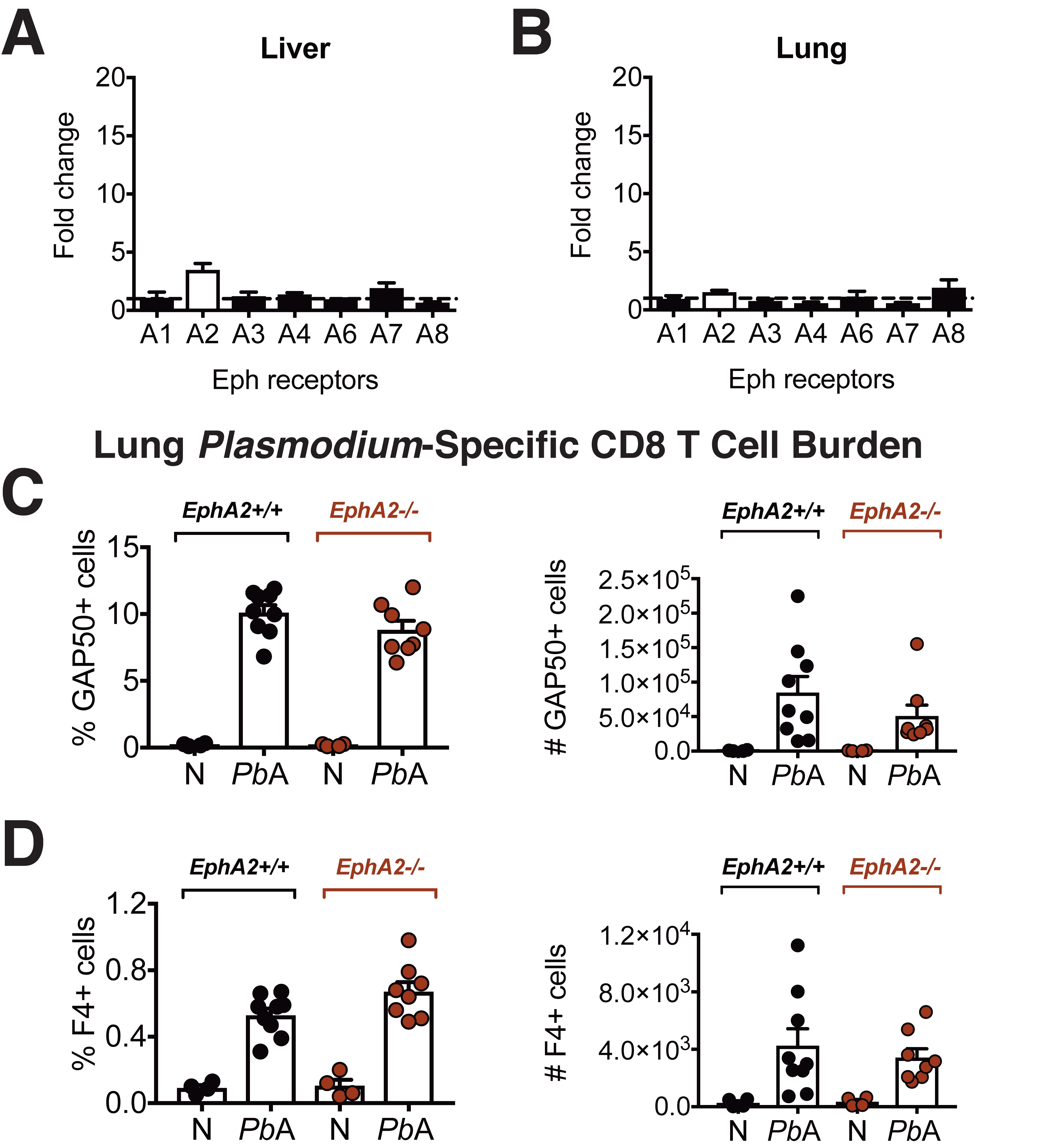

Supplement: S3 Fig — Transcription of EphA receptors relative to naïve mice (dashed line) in liver (n = 8/group) (A) and lung (n = 7-11/group) (B) lysates of C57BL/6J mice at day 6 post-infection with PbA. (C-D) Frequency (left) and total number (right) of Plasmodium GAP50-reactive (n = 8-9/group) (C) and Plasmodium F4-reactive (n = 8-9/group) (D) CD8+ T cells present in the lungs of EphA2-/- and littermate control mice at day 6 post-infection with PbA compared to naïve mice (N) (n = 4/group). Naïve and PbA-infected groups are significantly different within each genotype for all graphs. Bars in all graphs represent the mean ± SEM. Statistical analyses: Mann-Whitney tests (C-D). Only statistically significant (p<0.05) values are shown unless otherwise noted in the legend. Figures are representative of 2 (A-D) independent experiments. (TIF) [file ppat.1008261.s003.tif]

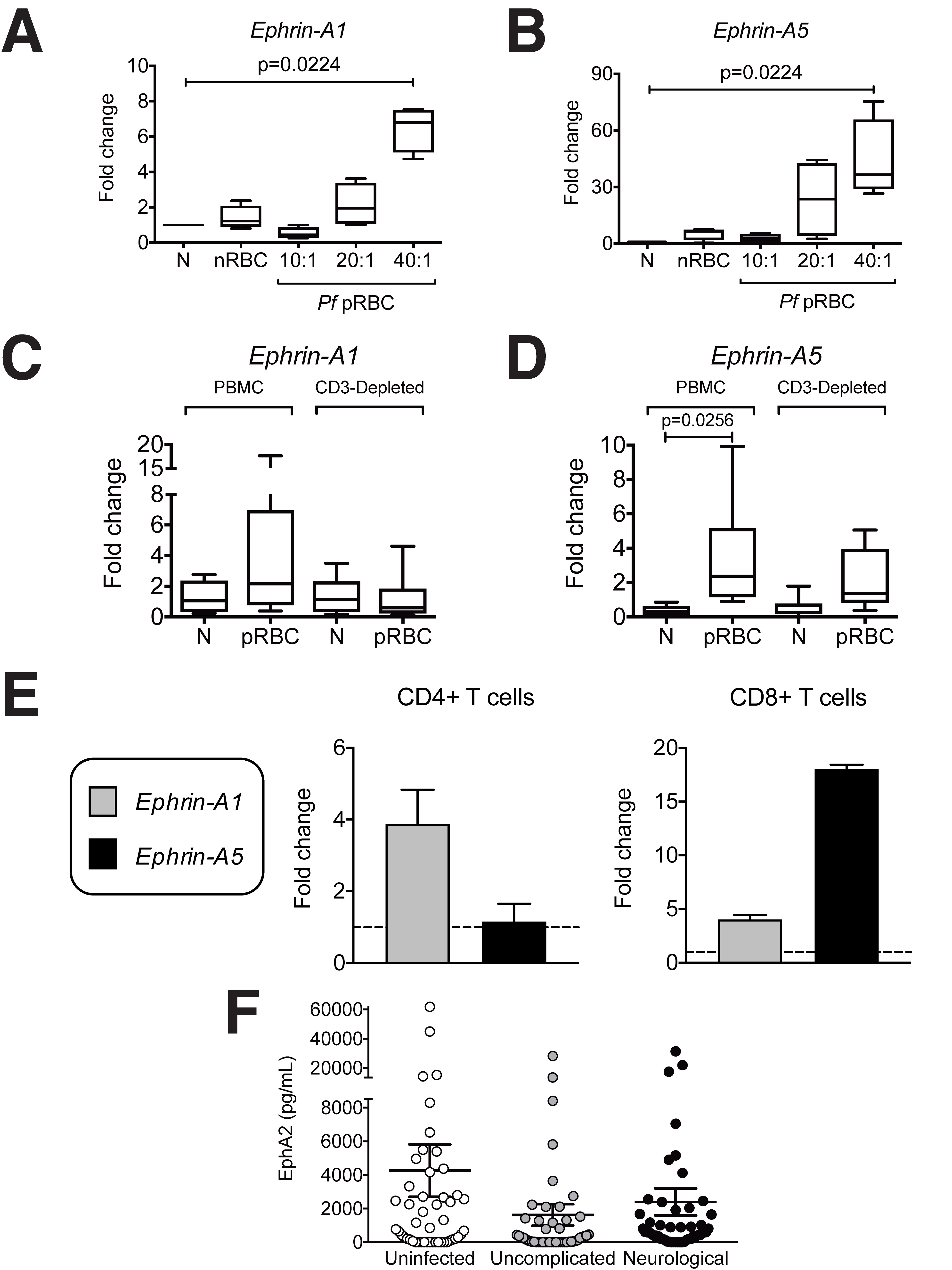

Supplement: S4 Fig — (A-B) Transcription of ephrin-A1 and ephrin-A4, ligands known to bind with high affinity to EphA2, in PBMCs isolated from healthy human donors incubated with naïve red blood cell lysates (nRBC) or P. falciparum-infected red blood cell lysates (Pf pRBC) (clone W2) at different ratios for 48 hours. (C-D) Transcription of ephrin-A1 and ephrin-A5 ligands in PBMCs isolated from healthy human donors incubated with naïve red blood cells lysates (nRBC) or P. falciparum-infected red blood cell lysates (pRBC) (clone 3D7) at a ratio of 40:1 for 48 hours before and after CD3+ T cell magnetic depletion. Boxes in A-D represent the median ±25th and 75th percentiles with minimum/maximum whiskers and transcription is relative to unstimulated PBMCs (N). (E) Transcription of ephrin-A1 and ephrin-A5 ligands on CD4+ and CD8+ T cells sorted from the spleens of C57BL/6J mice at day 5 post-infection with PbA (n = 8) relative to naïve mice (dashed line). (F) Levels of soluble EphA2 in the plasma of children living in an area in Cameroon endemic for P. falciparum malaria. Patients were categorized by admission to the hospital for neurological complications (n = 51), uncomplicated malaria (n = 50), or uninfected and presenting for routine pediatric tests (n = 49). Each dot represents an individual patient. Bars in E-G represent the mean ± SEM. Statistical analyses: Kruskal-Wallis and Dunn’s multiple comparisons tests (A-D) and General linear modeling and Tukey’s pairwise comparison post-ANOVA (G). Only statistically significant (p<0.05) values are shown. Figures are representative of 2 (E), 4 (A, B), or 6 (C, D) independent experiments. (TIF) [file ppat.1008261.s004.tif]

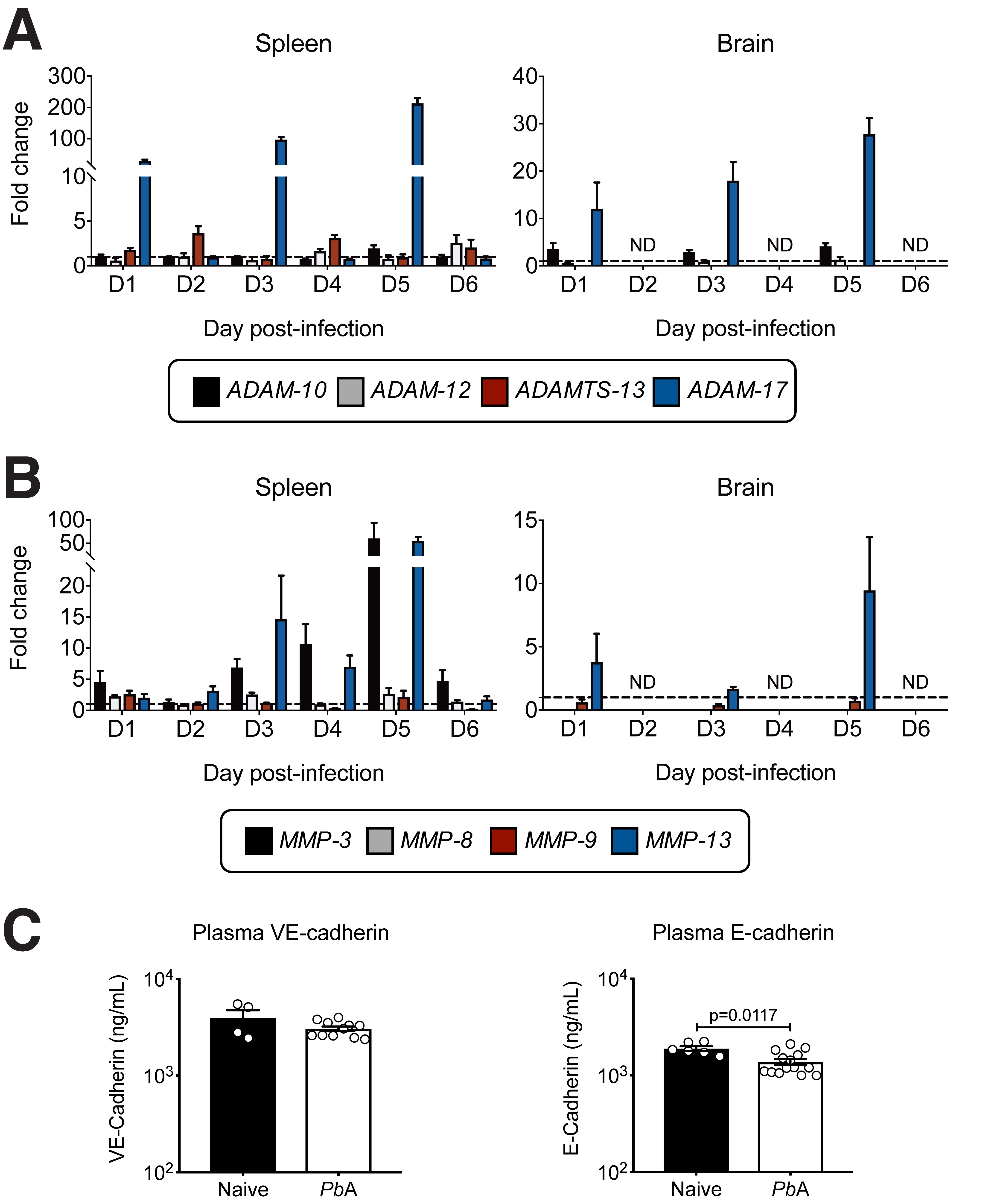

Supplement: S5 Fig — Transcription of a disintegrin and metalloproteinase domain-containing proteins (ADAM-10, ADAM-12, ADAM-17) with thrombospondin motifs (ADAMTS-13) (A) and matrix metalloproteinases (MMP-3, MMP-8, MMP-9, MMP-13) (B) relative to naïve mice (dashed line) in spleen (n = 4/day) and brain (n = 4/day) lysates of C57BL/6J mice at different time points post-infection with PbA. ND indicates no transcript was detected. (C) VE-cadherin (left) and E-cadherin (right) present in the plasma of C57BL/6J mice at day 6 post-infection with PbA (n = 11-14/group) compared to naïve mice (n = 4-6/group). Bars in all graphs represent the mean ± SEM. Statistical analyses: Mann-Whitney test (C). Only statistically significant (p<0.05) values are shown. Figures are representative of 1 (A-B) or 2 (C) independent experiments. (TIF) [file ppat.1008261.s005.tif]
