# Supplementary material for: EphA2 contributes to disruption of the blood-brain barrier in cerebral malaria
Source: PLoS Pathog. 2020 Jan 30;16(1):e1008261. doi: 10.1371/journal.ppat.1008261 (PMC6991964; doi:10.1371/journal.ppat.1008261)
Supplement: S1 Table — All mouse forward and reverse primer sequences used for mRNA amplification and RT-qPCR assays for the genes listed, including data found in both primary and supplemental figures, are included in this table. (DOCX) [file ppat.1008261.s006.docx]

**S1 Table**

| **Gene** | **Forward Primer** | **Reverse Primer** |
| --- | --- | --- |
| *Ephrin-A1* | CCTCAGGCCCATGACAATCCACAGG | CCTTCACGGGGTTTGCAGCAAGC |
| *Ephrin-A5* | CCAGGCGTGATGTTGCACGTGG | CCAGTAGACAGCGTAGCGGTCG |
| *EphA1* | ATCAGGGACTCAACTCTCCCT | TGCACACCTTTTCCACAGGGT |
| *EphA2* | TCCATTAAGGACTCGGGGCAGGAGG | CTCGCTCTCGGTCCGATCCCCC |
| *EphA3* | CCGCTCTGCTTCAGCGCACG | GGCTGCCTTTCCGCGAACCC |
| *EphA4* | GATGCCACCTGTGCCAAGTGCCC | TCAAGTTCAGGGGAGCAGATGGTGG |
| *EphA6* | ACTGTGGTGGATGCAATTCCCCTCG | TGCAGCTGCTTCGGAGGAGGACG |
| *EphA7* | CCAGAGGCTCTTCGCTGCTGT | CCGTGATGACTCCATTGGGAT |
| *EphA8* | CACCACGAACCAGGCAGCCC | CCACAGCAGCGAGACGCTGG |
| *Pb18S* | AAGCATTAAATAAAGCGAATACATCCT-TAC | GGAGATTGGTTTTGACGTTTATGTG |
| *CD8α* | GCTCAGTCATCAGCAACTCG | ATCACAGGCGAAGTCCAATC |
| *LT-α* | AGTCTGTGTATCCGGGACTTCAA | GGTCTCCCTTACTGAGCAGGAA |
| *TNF-α* | TCTCATTCCTGCTTGTGGC | CACTTGGTGGTTTGCTACG |
| *IL-6* | ACACATGTTCTCTGGGAAATCGT | AAGTGCATCATCGTTGTTCATACA |
| *CCL2* | GGCTCAGCCAGATGCAGTTAA | CCTACTCATTGGGATCATCTTGCT |
| *CCL3/MIP-1α* | CCATGACACTCTGCAACCAAGT | TCCGGCTGTAGGAGAAGCA |
| *CCL5/RANTES* | GCAAGTGCTCCAATCTTGCA | CTTCTCTGGGTTGGCACACA |
| *CXCL10* | GACGGTCCGCTGCAACTG | GCTTCCCTATGGCCCTCATT |
| *IFN-γ* | CGGCACAGTCATTGAAAGCCTA | GTTGCTGATGGCCTGATTGTC |
| *VE-cadherin* | CACTGCTTTGGGAGCCTTC | GGGGCAGCGATTCATTTTTCT |
| *HPRT* | GGCCCACCTAGTCAGATAAGAGTTCC | ATGGCTCAGAAACGCTGCCGG |
| *GAPDH* | TGTGTCCGTCGTGGATCTGA | TTGCTGTTGAAGTCGCAGGAG |
| *UBIQUITIN* | TGGCTATTAATTATTCGGTCTGCAT | GCAAGTGGCTAGAGTGCAGAGTAA |
| *MMP-3* | ACATGGAGACTTTGTCCCTTTTG | TTGGCTGAGTGGTAGAGTCCC |
| *MMP-8* | TCTTCCTCCACACACAGCTTG | CTGCAACCATCGTGGCATTC |
| *MMP-9* | CTGGACAGCCAGACACTAAAG | CTCGCGGCAAGTCTTCAGAG |
| *MMP-13* | CTTCTTCTTGTTGAGCTGGACTC | CTGTGGAGGTCACTGTAGACT |
| *ADAM-10* | TCATGGGTCTGTCATTGATGGA | TCAAAAACGGAGTGATCTGCAC |
| *ADAM-12* | TGGGACCAGAGAGGAGCTTAC | GTTGCACAGTCAGCACGTCT |
| *ADAMTS-13* | GAGGACACAGAACGCTACGTG | TTGGCCGTGATATTCGGAGTA |
| *ADAM-17* | GGAACACGTCGTGGGATAATG | GGCAGACTTTGGATGCTTCTT |
| *CLAUDIN-3* | GTACAAGACGAGACGGCCAA | CAGCCTAGCAAGCAGACTGT |
| *CLAUDIN-5* | GCAAGGTGTATGAATCTGTGCT | GTCAAGGTAACAAAGAGTGCCA |
| *OCCLUDIN* | TGAAAGTCCACCTCCTTACAGA | CCGGATAAAAAGAGTACGCTGG |

**S1 Table. Comprehensive list of mouse primers.** All mouse forward and reverse primer sequences used for mRNA amplification and RT-qPCR assays for the genes listed, including data found in both primary and supplemental figures, are included in this table.
