# Supplementary material for: EphA2 contributes to disruption of the blood-brain barrier in cerebral malaria
Source: PLoS Pathog. 2020 Jan 30;16(1):e1008261. doi: 10.1371/journal.ppat.1008261 (PMC6991964; doi:10.1371/journal.ppat.1008261)
Supplement: S2 Table — All human forward and reverse primer sequences used for mRNA amplification and RT-qPCR assays for the genes listed, including data found in both primary and supplemental figures, are included in this table. (DOCX) [file ppat.1008261.s007.docx]

| **Gene** | **Forward Primer** | **Reverse Primer** |
| --- | --- | --- |
| *Ephrin-A1* | CACAGTCCTCAGGCCCATGACAATCC | CGGGGTTTGCAGCAGCAGAAGTGG |
| *Ephrin-A5* | CCAGGCGTGATGTTGCACGTGG | CCAGTAGACAGCGTAGCGGTCG |
| *GAPDH* | GAGTCAACGGATTTGGTCGT | TTGATTTTGGAGGGATCTCG |
| *β-Actin* | CACGAAACTACCTTCAACTCC | CATACTCCTGCTTGCTGATC |

**S2 Table. Comprehensive list of human primers.** All human forward and reverse primer sequences used for mRNA amplification and RT-qPCR assays for the genes listed, including data found in both primary and supplemental figures, are included in this table.

**S2 Table**
